# TMEM219 signaling promotes intestinal stem cell death and exacerbates colitis

**DOI:** 10.1172/JCI185783

**Published:** 2025-05-15

**Authors:** Francesca D’Addio, Giovanni Amabile, Emma Assi, Anna Maestroni, Adriana Petrazzuolo, Cristian Loretelli, Ahmed Abdelasalam, Moufida Ben Nasr, Ida Pastore, Maria Elena Lunati, Vera Usuelli, Monica Zocchi, Andy Joe Seelam, Domenico Corradi, Stefano La Rosa, Virna Marin, Monique Zangarini, Marta Nardini, Stefano Porzio, Filippo Canducci, Claudia Nardini, Basset El Essawy, Manuela Nebuloni, Jun Yang, Massimo Venturini, Giovanni Maconi, Franco Folli, Silvio Danese, Gianvincenzo Zuccotti, Gianluca M. Sampietro, Sandro Ardizzone, Paolo Fiorina

**Affiliations:** 1 International Center for T1D, Pediatric Clinical Research Center Romeo ed Enrica Invernizzi, Department of Biomedical and Clinical Sciences, Università degli Studi di Milano, Milan, Italy.; 2 Division of Endocrinology, ASST Fatebenefratelli-Sacco, Milan, Italy.; 3 Enthera, Milan, Italy.; 4 Boston Children’s Hospital and Transplantation Research Center, Brigham and Women’s Hospital, Harvard Medical School, Boston, Massachusetts, USA.; 5 Department of Medicine and Surgery, Unit of Pathology, University of Parma, Parma, Italy.; 6 Department of Medicine and Surgery, Università degli Studi dell’Insubria, Varese, Italy.; 7 Nephrology Division, Brigham and Women’s Hospital, Harvard Medical School, Boston, MassachuseLs, USA.; 8 Department of Medicine, Al-Azhar University, Cairo, Egypt.; 9 Pathology Unit, ASST-Fatebenefratelli Sacco and Department of Biomedical and Clinical Sciences, Università degli Studi di Milano, Milan, Italy.; 10 Institute of Organ Transplantation, Tongji Hospital and Medical College, Huazhong University of Science and Technology, Wuhan, China.; 11 Diagnostic and Interventional Radiology Department, Circolo Hospital, ASST Sette Laghi and School of Medicine and Surgery, Università degli Studi dell’Insubria, Varese, Italy.; 12 Gastrointestinal Unit, ASST-Fatebenefratelli Sacco and Department of Biomedical and Clinical Sciences, Università degli Studi di Milano, Milan, Italy.; 13 Endocrinology and Metabolism, Department of Health Science, Università di Milano, Diabetic and Metabolic Diseases Unit-ASST Santi Paolo e Carlo, Milan, Italy.; 14 Gastroenterology and Endoscopy, IRCCS Ospedale San Raffaele and Medicine and Surgery Department, Vita-Salute San Raffaele University, Milan, Italy.; 15 Pediatric Clinical Research Center Romeo ed Enrica Invernizzi-Università di Milano and Buzzi Children’s Hospital, Milan, Italy.; 16 Division of General and HBP Surgery, Rho Memorial Hospital, ASST Rhodense, Milano, Italy.

**Keywords:** Gastroenterology, Stem cells, Apoptosis, Inflammatory bowel disease, Mouse models

## Abstract

Mechanisms by which mucosal regeneration is abrogated in inflammatory bowel disease (IBD) are still under investigation, and a role for an intestinal stem cell (ISC) defect is now emerging. Herein, we report an abnormal ISC death that occurs in Crohn’s disease, which exacerbates colitis, limits ISC-dependent mucosal repair, and is controlled through the death factor Transmembrane protein 219 (TMEM219). Large alterations in TMEM219 expression were observed in patients with Crohn’s disease, particularly in those with active disease and/or those who were nonresponders to conventional therapy, confirming that TMEM219 signaling is abnormally activated and leads to failure of the mucosal regenerative response. Mechanistic studies revealed a proapoptotic TMEM219-mediated molecular signature in Crohn’s disease, which associates with Caspase-8 activation and ISC death. Pharmacological blockade of the IGFBP3/TMEM219 binding/signal with the recombinant protein ecto-TMEM219 restored the self-renewal abilities of miniguts generated from patients with Crohn’s disease in vitro and ameliorated DSS-induced and T cell-mediated colitis in vivo, ultimately leading to mucosal healing. Genetic tissue-specific deletion of TMEM219 in ISCs in newly generated TMEM219^fl/fl^LGR5^cre^ mice revived their mucosal regenerative abilities both in vitro and in vivo. Our findings demonstrate that a TMEM219-dependent ISC death exacerbates colitis and that TMEM219 blockade reestablishes intestinal self-renewal properties in IBD.

## Abstract 2

Our study showed that a TMEM219-dependent intestinal stem cells death exacerbates colitis and that TMEM219 blockade re-establishes intestinal self-renewal properties in inflammatory bowel disease.

## Graphical abstract

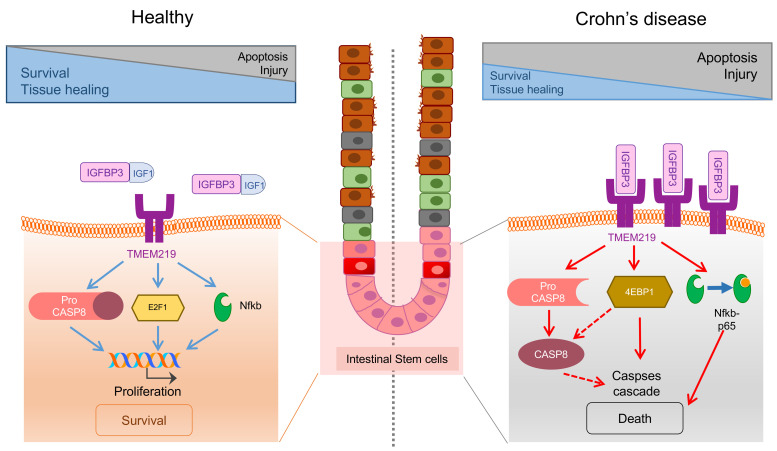

## Introduction

Chronic inflammation of the small and large intestines associated with acute flares and remitting symptoms are key features of inflammatory bowel disease (IBD), particularly Crohn’s disease (CD) (1, 2). Over the past 2 decades, suppression of immune and inflammatory responses has represented a cornerstone in the medical therapy of CD; however, the relapsing-remitting nature of the disease still leads to unsuccessful clinical results (3–5). This is possibly due to the lack of strategies that could harness intestinal mucosa regeneration (6–8). Recent studies have suggested that intestinal stem cell (ISC) apoptosis replenishment of the mucosa is altered in CD, leading to disruption of tissue regeneration (3, 9). Impairment of ISCs, which limits the mucosal response to damage, has also been described in other gastrointestinal disorders, such as colorectal cancer (10) and environmental colitis (infectious, toxic, drug induced, or disease associated) (11–15). Several cell death mechanisms and factors, including tumor necrosis factor-α (TNF-a) or CD95 ligand, regulate epithelial cell turnover in the intestine and act by activating Caspase-8 cascade (16, 17). Recently, a Caspase-8–dependent cell death/survival mechanism has been described for the death receptor Transmembrane protein 219 (TMEM219) (18, 19) when binding to its ligand, the insulin-like growth factor binding protein 3 (IGFBP3) (20, 21), and a TMEM219 signaling has been also described in ISCs (22). Despite being the major carrier for insulin-like growth factor-1 (IGF-I) in the circulation and an important regulator for its bioavailability to tissues and organs, IGFBP3, the most abundant among the 6 IGFBPs in the bloodstream, can also exert an IGF-I–independent effect in different cell types (18, 23, 24) and particularly in ISCs, by signaling through TMEM219 and favoring ISCs loss and dysfunction (22). Whether abnormalities in the compartment and function of ISCs exist in CD, which lead to an uncontrolled destruction and inflammation of the intestinal mucosa and may halt the healing of the tissue, has only been partially explored. In this study, we demonstrated the existence of an abnormal ISC death in CD, which exacerbates colitis, limits ISC-dependent mucosal repair, and is controlled through the death factor TMEM219. Indeed, TMEM219-mediated ISC dysfunction occurs through activation of Caspase-8, and it is associated with failing regenerative abilities of the intestinal mucosa and persistent inflammation. Genetic and pharmacological blockade of the IGFBP3/TMEM219 signaling successfully preserved ISCs and their self-renewal properties in vitro, enabled proliferation and recovery of intestinal crypts, and ameliorated the signs and symptoms of acute and chronic colitis in preclinical models in vivo.

## Results

### ISC defects exist in CD.

**Figure 1:**
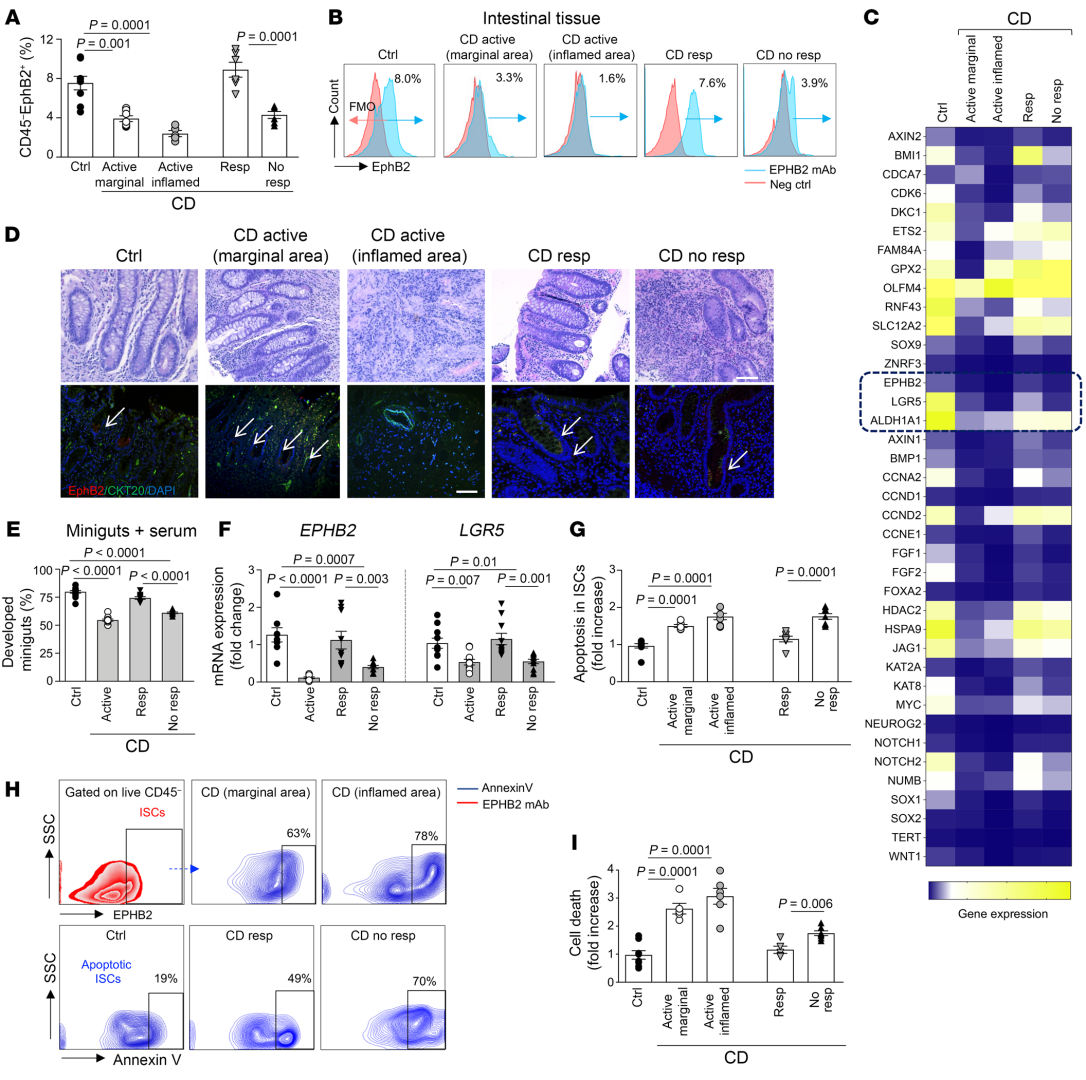
Intestinal stem cell defects exist in active Crohn’s disease. (**A** and **B**). Flow cytometric analysis of CD45^–^EPHB2^+^ ISCs of patients with active Crohn’s disease (CD, marginal and inflamed areas), responders in remission phase, nonresponders, or controls (*n* = 6–10/group). (**C**). Transcriptome profile delineating the stem cell–related signature in intestinal samples of controls and of patients with CD from all patient cohorts. (**D**). Representative pictures of H&E (upper panels) and confocal analysis (lower panels) of the ISC marker EPHB2 (red) and of the epithelial marker Cytokeratin 20 (CKT20, green) in intestinal samples of controls and of patients with Crohn’s disease from all patient cohorts. Nuclei stained by DAPI. Original magnification ×20; scale bar: 100 μm. (**E**). Development of miniguts from crypts isolated from controls and grown in pooled serum of people who were controls or patients (*n* = 5) with Crohn’s disease from all patient cohorts, in place of 10% FBS (*n* = 10/group). (**F**). Normalized mRNA expression of the ISC markers *EPHB2* and *LGR5* quantified in miniguts, as described in **E** (*n* = 10/group). (**G** and **H**). Flow cytometric analysis (fold increase) and flow plots of Annexin-V^+^ ISCs of patients with Crohn’s disease from all patient cohorts compared with people who were controls (*n* = 7–9/group). (**I**). Cell death analysis in intestinal samples of people who were controls and of patients with Crohn’s disease from all patient cohorts (*n* = 5–8/group). Mean ± SEM. At least 3 independent experiments performed in duplicate. 1-way ANOVA followed by Šidák’s post hoc analysis.

To determine whether ISCs are altered in CD, we first explored the expression of ISC markers by transcriptomic, flow cytometry, and IF confocal analyses in intestinal tissue samples of patients with CD in different disease stages (Supplemental Table 1; supplemental material available online with this article; https://doi.org/10.1172/JCI185783DS1) compared with patients without CD, who were considered controls. Our flow cytometry analysis demonstrated a decrease of ISCs (CD45^–^EphB2^+^) in samples of patients with active disease obtained from the marginal area and absence of ISCs nearby the inflammatory lesion (inflamed area), (Figure 1, A and B and Supplemental Figure 1A). Few ISCs were also detected in patients with disease not responding to conventional first-line or second-line therapy (nonresponders), while ISCs appeared more abundant in patients responding to therapy in the remission phase (responders), thus suggesting the existence of a defect in ISCs, particularly in active disease (Figure 1, A and B). A stem cell transcriptome profile also revealed a decreased expression of the most relevant stem cell markers in samples of patients with active disease, primarily in the inflamed area, and in patients who were nonresponders, including the ISC markers *EPHB2* and *LGR5,* whose expression was near normalized in patients who were responders in remission phase (Figure 1C and Supplemental Table 2). A confocal analysis further confirmed decreased expression of the ISC marker EPHB2 in patients with active disease and in nonresponders with no detectable expression in the inflamed area, in which the crypt architecture was completely subverted and destroyed (Figure 1D and Supplemental Figure 1B). ISH also demonstrated a decreased LGR5 positivity in patients with active disease compared with individuals who were controls, thereby supporting the existence of an ISC defect in active CD (Supplemental Figure 1C). The reappearance of ISCs observed in responder patients (Figure 1, A–D) strengthened the relevance of an ISC-mediated mucosal repair in CD. To next demonstrate a functional defect of ISCs in CD, we generated large crypt organoids, namely “miniguts”, from all patient cohorts with CD and from patients without CD. The development of miniguts, with at least 1 crypt domain visible after an 8 day culture, was reduced up to 50% in patients with active disease (marginal area) and in patients who were non-responders compared with individuals who were controls, with only spheroids developing from the inflamed area (Supplemental Figure 1, D–F). The parallel reduction observed in the mRNA expression of the ISC markers *EPHB2* and *LGR5* in these miniguts pointed at a defect in ISCs primarily responsible for the failure of mucosal regeneration (Supplemental Figure 1, D–F); this was further confirmed by demonstrating a recovery of self-renewal ability and ISC marker expression in miniguts of patients who were responders in remission phase. In line with this, organoids generated from crypts of participants who were controls cultured in the presence of pooled sera of patients with active disease and of patients who were nonresponders failed to normally grow compared with those cultured with control serum or with serum of responder patients in remission phase (Figure 1E) and showed low expression of ISC markers (Figure 1F). We finally confirmed that the defects in ISCs in CD also reside in their prone-to-death characteristics, as the ISCs of patients with active CD, particularly those extracted from the inflamed area, and of patients who were nonresponders were highly apoptotic (Figure 1, G and H) and had a high rate of dying cells (Figure 1I) compared with ISCs from people who were controls and patients who were responders in remission phase. These findings demonstrate that a defect in ISC expression and function exists in active CD and may be responsible for the inability of intestinal mucosa to regenerate and respond to inflammatory and environmental triggers.

### Dysfunctional Caspase-8–mediated TMEM219 signaling is present in CD.

**Figure 2:**
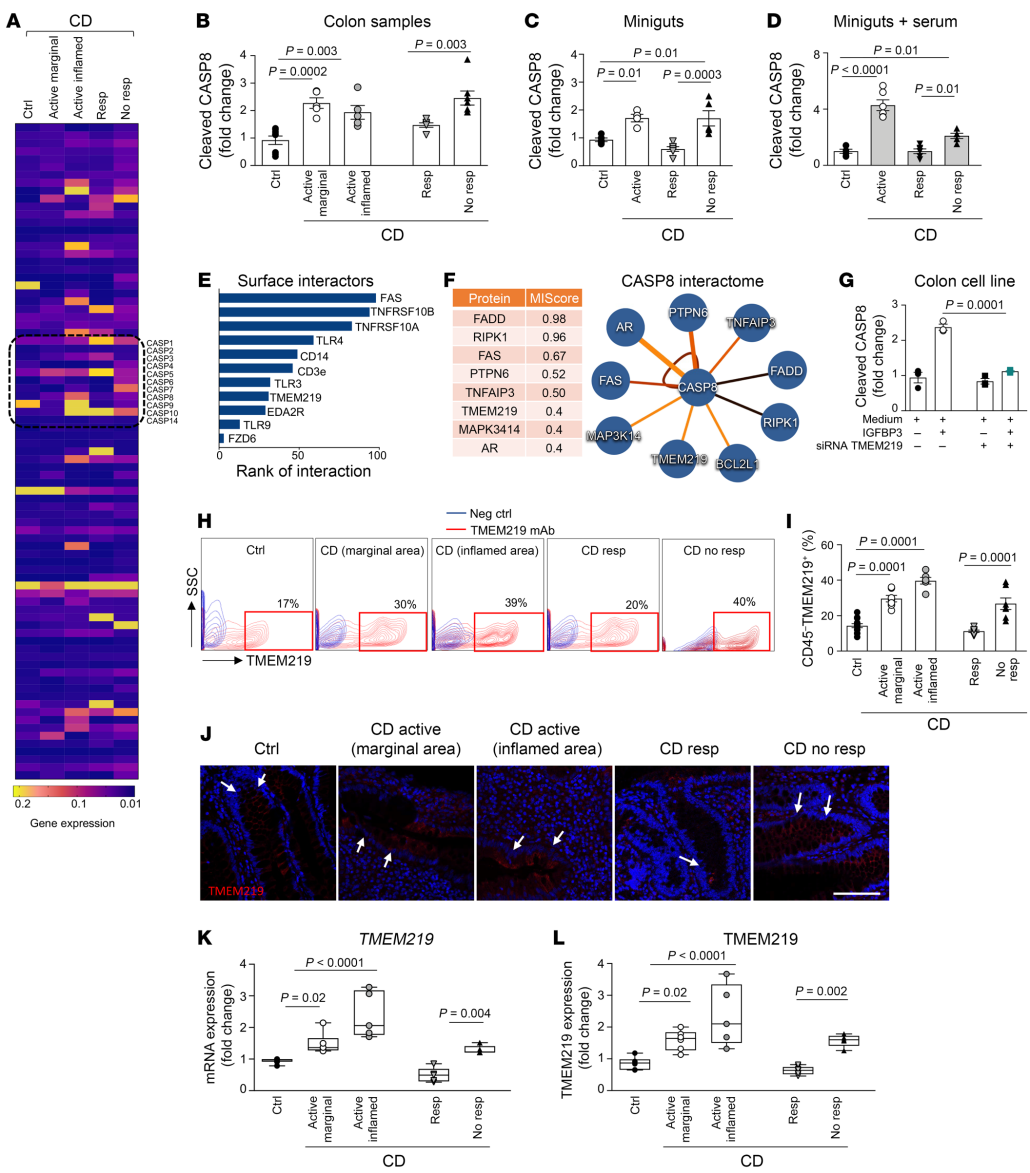
Dysfunctional Caspase-8–mediated TMEM219 signaling in Crohn’s disease. (**A**). Apoptotic gene signature analyzed in intestinal samples of patients with active Crohn’s disease (CD, marginal and inflamed areas), of patients who were responders in remission phase and nonresponders compared with controls (Ctrl). (**B**). Cleaved Caspase-8 measured in intestinal samples of people who were controls and of patients with Crohn’s disease from all patient cohorts (*n* = 5–8/group). (**C** and **D**). Cleaved Caspase-8 quantified in miniguts developed from individuals who were controls and from patients with Crohn’s disease of all patient cohorts (*n* = 6/group) or measured in miniguts of control samples cultured with pooled serum of people who were controls or patients with Crohn’s disease (*n* = 5) from all patient cohorts in place of 10% FBS (*n* = 6/group). (**E**). Cell membrane receptors identified in the Caspase-8 interactome and depicted based on their ranking of interactions using Genemania analysis. (**F**). Network of Caspase-8 gene-gene interactions generated by the IntAct software, based on molecular interaction, type, and method of detection. Top 8 genes and MIscore for interaction with Caspase-8 are shown. (**G**). Cleaved Caspase-8 measured in Caco2 cells transfected with siRNA TMEM219 and cultured with the TMEM219 ligand IGFBP3 (50 ng/mL), (*n* = 3). (**H** and **I**). Flow cytometric expression of TMEM219 in intestinal cells of people who were controls (*n* = 13) and patients with Crohn’s disease from all patient cohorts (*n* = 7/group). (**J**). Representative pictures of TMEM219 immunofluorescence expression in intestinal samples of people who were controls and of patients with Crohn’s disease from all patient cohorts. The crypt base location of positive cells is highlighted. Original magnification ×20; scale bar: 100 μm. (**K** and **L**). TMEM219 protein and mRNA expression quantified in intestinal samples of patients with Crohn’s disease from all patient cohorts compared with controls (*n* = 5–8). Mean ± SEM. Box plots include the median line, the minimum and maximum value, and the upper and lower quartile. At least 3 independent experiments performed in duplicates. 1-way ANOVA followed by Šidák’s post hoc test and 2-sided *t* test.

Given that ISCs appeared to be more prone to death in CD, we explored the apoptosis/cell death transcriptome in samples of patients with active CD, samples of marginal and inflamed areas from patients with active CD, in patients who were responders in remission phase, and in patients who were nonresponders compared with people who were controls. We delineated a proapoptotic signature in active disease, with several proapoptotic factors overexpressed and with *CASP8* being a major factor linked to disease activity and response to therapy (Figure 2A and Supplemental Table 3). To better address the role of Caspase-8, we next analyzed Caspase-8 activation and demonstrated increased levels of Cleaved Caspase-8 in active disease and in patients who were nonresponders, while a decrease to normal was observed in patients who were responders in remission phase (Figure 2B). Confirmatory analyses in intestinal organoids, miniguts, generated from patients with CD at different disease stages and in miniguts cultured in the presence of serum of all patient cohorts revealed a 2-to-3–fold increase in cleaved Caspase-8 in patients who were in active disease/nonresponding to therapy (Figure 2, C and D) compared with individuals who were controls and to patients who were responders in remission phase (Figure 2, C and D). These results suggest a link between the apoptotic signature of ISCs and the failure of self renewal in active CD (Figure 2, C and D). Using a bioinformatics approach and available databases (Genemania Cytoscape [https://genemania.org/], IntAct-EMBL-EMI [https://www.ebi.ac.uk/intact/documenta:on/user-guide], and PINA 3.0 [https://omics.bjcancer.org/pina/]), we delineated the Caspase-8 interactome (Supplemental Figure 1, G and H and Supplemental Table 4). After excluding all the intracellular transcription factors and other proapoptotic transcripts, we focused on surface molecules (Figure 2E) and observed that TMEM219 appeared to be strictly associated as a major interactor and partner for the Caspase-8 signaling pathway (Figure 2F and Supplemental Figure 1I). In vitro mechanistic studies further demonstrated that TMEM219 silencing through siRNA prevented Caspase-8 activation (Figure 2G), and that direct inhibition of the TMEM219 signaling correlated with levels of activated Caspase-8 in a dose-dependent manner (Supplemental Figure 2A). Based on the observation that a Caspase-8–mediated ISC defect exists and that it may be linked to overactive TMEM219 signaling, we moved to explore whether TMEM219 expression was altered in intestinal samples of our patient cohorts. A flow cytometric analysis first confirmed that TMEM219 was expressed in intestinal cells and demonstrated an elevated percentage of TMEM219-positive cells in patients with active CD and in patients who were nonresponders, while TMEM219-expressing cells were fewer in patients who were responders in remission phase, similar to controls (Figure 2, H and I). This observation was paralleled in an immunofluorescence confocal analysis, which also revealed that TMEM219 positivity was mainly, although not exclusively, located at the base of the crypt, where ISCs and progenitor cells reside, and that TMEM219 was coexpressed with the stem cell marker Aldehyde Dehydrogenase and with the ISC marker LGR5 (Figure 2J and Supplemental Figure 2, B and C). This was also proved in organoids derived from controls, while the lack of LGR5 expression did not allow the same observation in active CD (Supplemental Figure 2D). Moreover, TMEM219 was only slightly detectable in other intestinal epithelial subtypes such as enterocytes and enteroendocrine cells (Supplemental Figure 2E). Finally, an analysis of TMEM219 mRNA and protein levels confirmed an elevated TMEM219 expression in active disease and in non-responder patients as compared to controls and to responder patients in the remission phase, in which TMEM219 level returned to normal (Figure 2, K and L). The aforementioned data also matched with a peripheral proinflammatory cytokine profile observed in active disease (Supplemental Figure 2F) and suggested a role for the local inflammatory environment in mediating TMEM219 upregulation. Increased TMEM219 expression was primarily evident in intestinal cells cultured with inflammatory cytokines and it was associated with higher cell death. Further analysis also demonstrated that upregulated TMEM219 expression was also detectable in presence of other noxae (i.e., ER and oxidative stress or increased local IGFBP3), resulting in an increased cell death (Supplemental Figure 3, A and B). Indeed, IGFBP3 is highly expressed in the liver of patients with active disease, and it is highly produced in vitro by hepatocytes cultured with TNF-a (Supplemental Figure 3, C and D). Our results suggest that an abnormal Caspase-8–mediated TMEM219 signaling is active in CD within the intestinal mucosa and may halt tissue regeneration and healing.

### Mechanistic studies delineate TMEM219-related proapoptotic downstream signaling.

**Figure 3:**
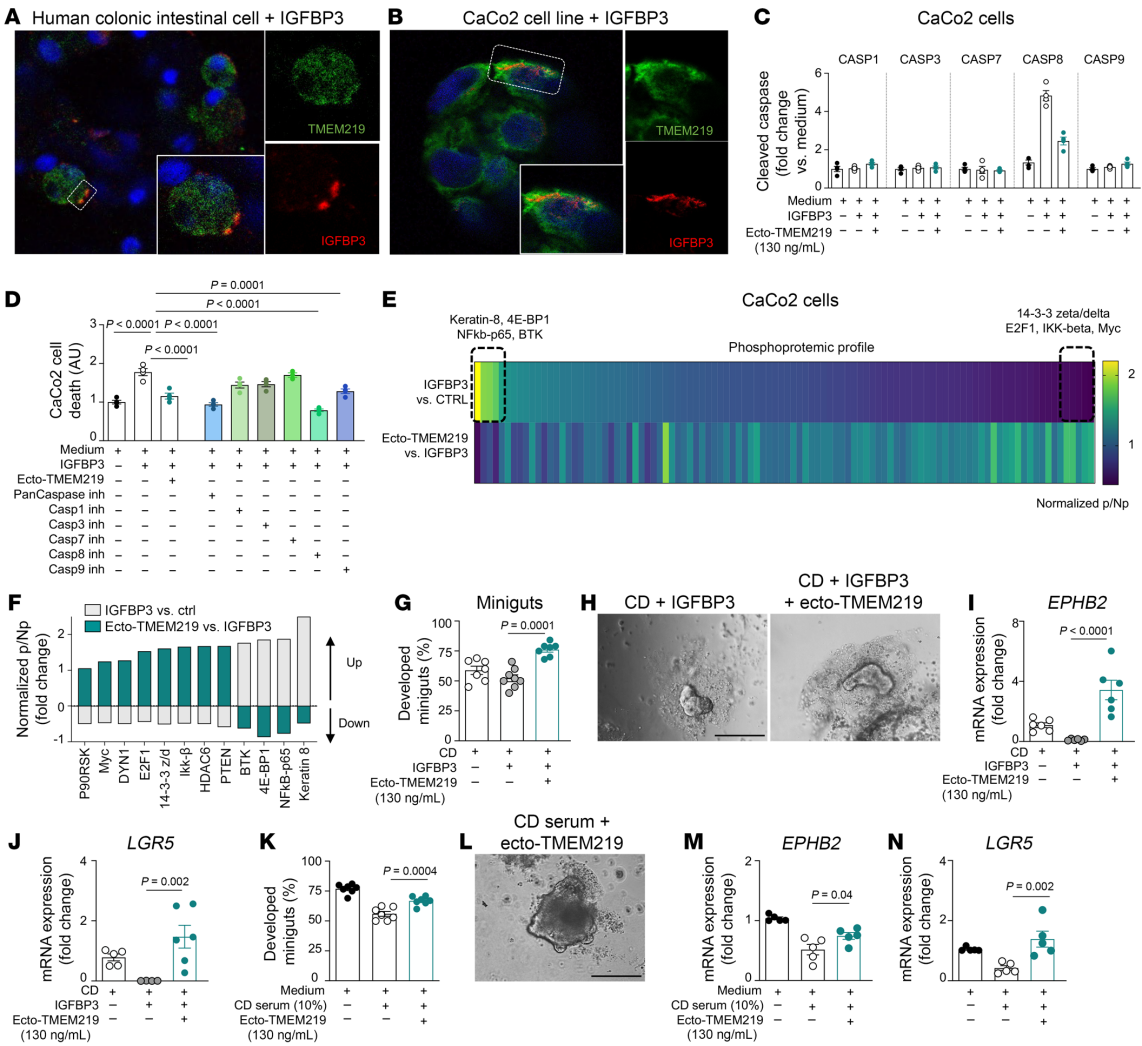
Mechanistic studies delineate a TMEM219-related proapoptotic downstream signaling. (**A** and **B**). Confocal microscopy analysis (Scale bar: 10 μm; original magnification ×63) depicting colocalization and binding of TMEM219 (green) and IGFBP3 (red) in intestinal cells dissociated from a control sample and incubated with recombinant IGFBP3 overnight and in CaCo2 cells. Cells were stained with DAPI for nuclei (blue) and immunolabeled with anti-TMEM219 (green) and anti-IGFP3 Abs (red). (**C**). Cleaved/activated Caspases 1, 2, 3, 7, 8, and 9 were quantified in CaCo2 cells cultured with/without IGFBP3 (50 ng/mL) and with/without the TMEM219 inhibitor ecto-TMEM219 (newly generated recombinant protein based on the TMEM219 extracellular portion). (**D**). Cell death quantified in CaCo2 cells cultured with/without IGFBP3 with ecto-TMEM219, Pan-caspase inhibitor, and selective inhibitors for Caspases 1, 3, 7, 8, and 9. (**E** and **F**). Phosphoproteomic profile identified in CaCo2 cells cultured with/without IGFBP3 (50 ng/mL) and with/without ecto-TMEM219 (130 ng/mL). Differentially expressed phosphorylated proteins (normalized to control) are presented in the heatmap as a ratio between nonphosphorylated and phosphorylated protein (mean value). In **F**, up/downregulated phosphorylated proteins with IGFBP3 and with Ecto-TMEM219 are reported. (**G** and **H**). Development of miniguts obtained from crypts of patients with active Crohn’s disase (CD) and cultured with/without IGFBP3 and ecto-TMEM219 (*n* = 7). Original magnification ×20; scale bar: 100 μm. (**I** and **J**). Normalized mRNA expression of *EPHB2* (**I**) and *LGR5* (**J**) in miniguts as described in **G** (*n* = 6). (**K** and **L**). Development of miniguts obtained from crypts of controls (Ctrl) and cultured in the presence of pooled sera of patients with active CD in place of 10% FBS and with/without ecto-TMEM219 (*n* = 7). Original magnification ×20; scale bar: 100 μm. (**M** and **N**). Normalized mRNA expression of *EPHB2* (**M**) and *LGR5* (**N**) in miniguts obtained as reported in **K** (*n* = 5). Mean ± SEM. At least 3 independent experiments run in duplicate. 1-way ANOVA followed by Šidák’s post hoc test and 2-sided *t* test.

To demonstrate the potential benefit of blocking TMEM219 signaling in target cells, such as intestinal progenitors/stem cells, we first demonstrated the binding between IGFBP3 and TMEM219-expressing cells within purified intestinal cells obtained from individuals who were controls (Figure 3A). Next, we confirmed in vitro, using the intestinal cell line CaCo2, that TMEM219 is expressed on the cell surface and it binds both to the IGFBP3 protein and to IGFBP3 circulating in blood (Figure 3B and Supplemental Figure 4, A and B). As the activation of TMEM219-mediated cell death is thought to occur through the Caspase-8 pathway, we confirmed that, among all the Caspases potentially involved in the downstream signaling, only activation of Caspase-8, measured through cleaved Caspase-8 quantification, was increased in intestinal cells cultured in the presence of the TMEM219 ligand IGFBP3 (50 ng/mL), (Figure 3C). As a proof of mechanism, when all the Caspase inhibitors were tested in the same experiment, Pan-caspase and Caspase-8–selective inhibitors were the most powerful in blocking the TMEM219 death signal (Figure 3D). To this end, we also demonstrated that, in the miniguts assay, in which we cultured organoids generated from individuals who were controls with pooled sera of patients with active disease, the Caspase-8–selective inhibitor and the Pan-caspase inhibitor to a less extent, among all inhibitors, preserved organoid development and prevented ISC death (Supplemental Figure 4, C and D). Moreover, the activation of TMEM219 deleterious signaling in target cells was confirmed by a reduction in the activated prosurvival factor AKT (Supplemental Figure 4E). Indeed, a phosphoproteomic analysis in intestinal cells cultured in the presence of IGFBP3 demonstrated decreased activation of antiapoptotic factors primarily involved in cell cycle regulation (i.e., Ikkb and Myc), an increased activation of proapoptotic mediators, particularly of eukaryotic initiation factor 4E–binding protein 1 (eIF4E-BP1), involved in Caspase-mediated cell apoptosis, and of NFkb-p65, which blocks the NFkb pathway, thereby suggesting that the TMEM219 signal may also promote cell apoptosis by disrupting cell cycle regulation (Figure 3E). Interestingly, phosphoproteomic analysis also revealed that blocking TMEM219 signaling with the recombinant protein ecto-TMEM219, which prevents IGFBP3 interaction with TMEM219, triggered some positive and prosurvival pathways (Figure 3F). Moreover, ecto-TMEM219 abrogated the activation of apoptotic mediators 4E-BP1, BTK, and NFkb-p65, which further blocked the detrimental effect of TMEM219 and protected intestinal cells from death (Figure 3F). Therefore, we tested whether pharmacological blockade of the IGFBP3/TMEM219 signaling/binding with ecto-TMEM219 in vitro could protect ISC function from IGFBP3-mediated negative effects. We first demonstrated that development of miniguts derived from healthy controls decreased in the presence of IGFBP3 and was rescued primarily by ecto-TMEM219, while addition of IGF-I, the IGFBP3 ligand, at increasing concentrations, did not show any effect (Supplemental Figure 4F) nor did it prevent intestinal cell death or Caspase 8 activation (Supplemental Figure 4, G and H). Interestingly, miniguts failed to grow when crypts were obtained from patients with active CD, and the addition of ecto-TMEM219 to the culture medium restored the self-renewal abilities of the crypts, with an increased development of near-normal organoids (Figure 3, G and H). This was associated with an increase in the ISC marker mRNA expression of *EPHB2* and *LGR5,* and with a downregulation of the proapoptotic TMEM219-related factor *CASP8* (Figure 3, I and J and Supplemental Figure 4I). Notably, development of organoids derived from patients with active CD were not further reduced in the presence of IGFBP3 in the culture. We demonstrated an increased local IGFBP3 expression in intestinal samples of patients with active disease compared with controls, which may suggest an already overactive TMEM219 signaling (Supplemental Figure 4J). Moreover, the development of miniguts cultured in the presence of serum of patients with active CD was reduced compared with those cultured with control serum, whereas ecto-TMEM219 nearly normalized their growth (Figure 3, K and L). The beneficial effect of IGFBP3/TMEM219 signaling blockade in this model was proven by the recovery of ISC marker expression (Figure 3, M and N) and was associated with a decrease in *CASP8* and in ISC cell death (Supplemental Figure 3D and Supplemental Figure 4K). Our results demonstrate that blocking TMEM219 deleterious signaling in vitro restores intestinal self-renewal abilities in organoids generated from patients with CD.

### Pharmacological blockade of IGFBP3/TMEM219 signal ameliorates DSS-mediated acute and chronic colitis in vivo.

**Figure 4:**
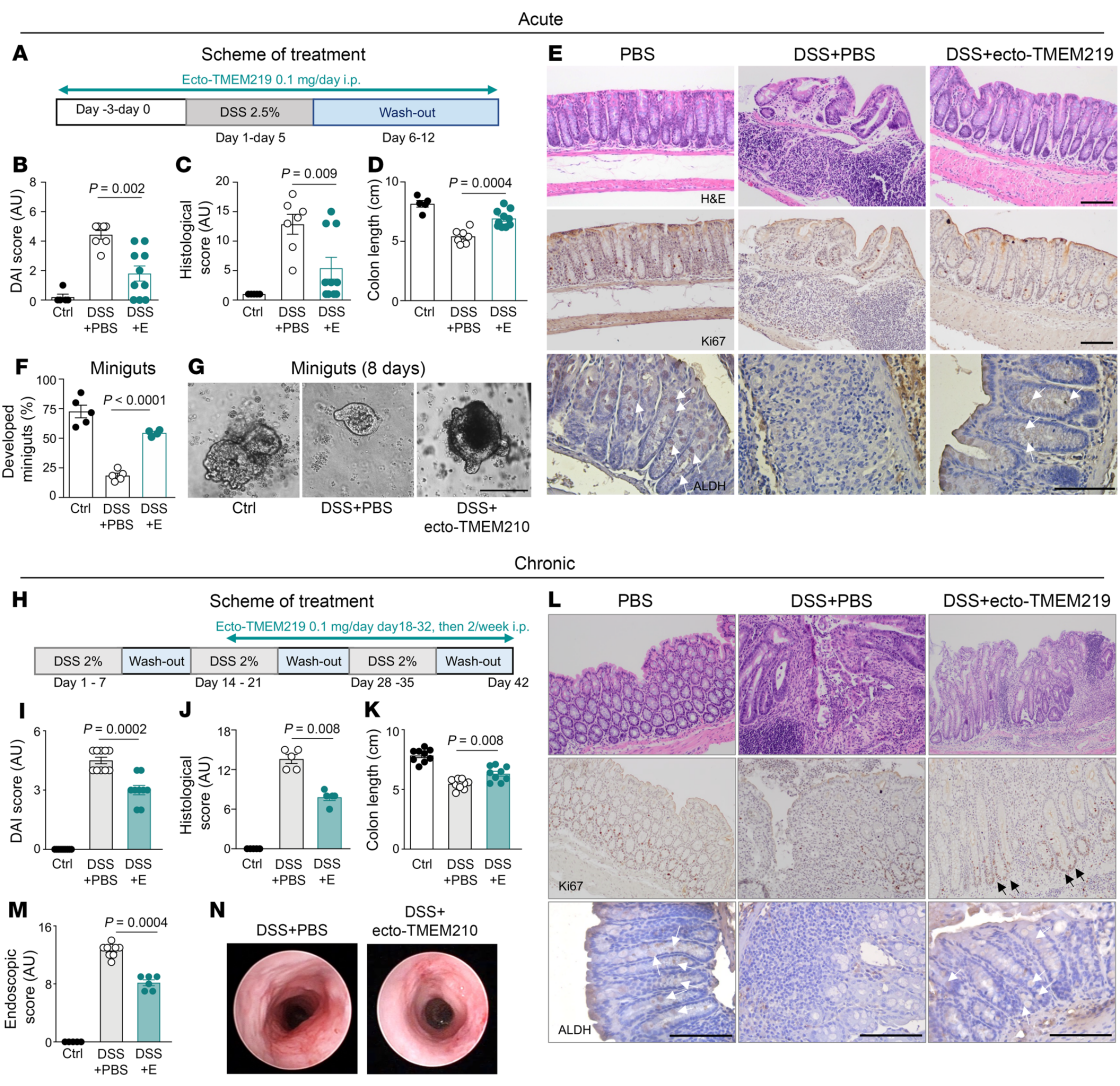
Pharmacological blockade of IGFBP3/TMEM219 signal ameliorates DSS-mediated acute and chronic colitis in vivo. (**A**). Experimental design of the DSS acute prevention model. (**B**–**D**). Disease activity index (DAI), histological score, and colon length measured at day +12 in control (*n* = 5), DSS+PBS and DSS+ecto-TMEM219–treated mice (*n* = 7–10). (**E**). Representative pictures of H&E staining, crypt proliferation (MKI67), and ALDH immunostaining in colons of controls, DSS+PBS, and DSS+ecto-TMEM219–treated mice. Original magnification ×20 (upper and middle panels), ×40 (lower panels); scale bars: 100 μm. Arrows indicate ALDH^+^ cells (lower panels). (**F** and **G**). Development of 8-day miniguts obtained from colons of controls, DSS+PBS, and DSS+ecto-TMEM219–treated mice (*n* = 5/group). Original magnification ×20; scale bar: 100 μm. (**H**). Experimental design of the DSS chronic treatment model. (**I**–**K**). DAI (*n* = 10), histological score (*n* = 5), and colon length (*n* = 10) measured at day 42 in controls, DSS+PBS, and DSS+ecto-TMEM219–treated mice. (**L**). Representative pictures of H&E staining, crypt proliferation (MKI67), and ALDH immunostaining in colons of controls, DSS+PBS, and DSS+ecto-TMEM219–treated mice. Original magnification ×20 (upper and middle panels) and ×40 (lower panels); scale bar: 100 μm. Arrows indicate MKI67^+^ cells (middle panel) and ALDH^+^ cells (lower panels). (**M** and **N**). Colon endoscopic analysis in controls, in mice receiving DSS+PBS (*n* = 9),or DSS+ecto-TMEM219 (*n* = 6), (Day 42). Mean ± SEM. 1-way ANOVA followed by Šidák’s post hoc test and 2-sided *t* test.

Next, we used in vivo models to determine whether pharmacological blockade of the IGFBP3/TMEM219 signaling pathway results in the preservation of mucosal regenerative abilities and protects the intestine from damage. We used a Dextran Sodium Sulfate–induced (DSS-induced) acute colitis mouse model, in which DSS was orally administered for 5 days, and ecto-TMEM219 was administered starting at day –3 in a preventive approach (Figure 4A). Interestingly, animals treated with ecto-TMEM219, compared with those treated with PBS, showed lower weight loss (Supplemental Figure 5A) and had less diarrhea and nearly absent occult blood/rectal bleeding, which resulted in a reduced disease activity index (DAI) score at the end of the study (Figure 4B). Colon length was well preserved in ecto-TMEM219–treated animals and associated with remarkably reduced leukocyte infiltration, which led to a significant improvement in the histological score (Figure 4, C and D and Supplemental Figure 5B). Notably, MKI67 immunostaining, which marks colonic proliferation and was nearly undetectable in animals receiving the DSS + PBS, was clearly detectable in the intestines of animals treated with ecto-TMEM219, suggesting highly proliferating crypts with near-normalized morphology, paralleling the features observed in naive untreated animals (Figure 4E). This was further confirmed by observing positive ALDH immunostaining in the intestinal crypts of ecto-TMEM219–treated animals (Figure 4E) and by the demonstration of a rescue of self-renewal abilities in ex vivo–generated miniguts compared with those obtained from DSS+PBS–treated mice, which failed to grow and develop (Figure 4, F and G). Restoration of the regenerative abilities of the intestinal mucosa was also demonstrated by the reestablishment of ISC markers *Lgr5* and *EphB2* mRNA expression, with *Casp8* being significantly downregulated (Supplemental Figure 5, C and E). We also confirmed the effect of pharmacological blockade of the IGFBP3/TMEM219 signaling/binding in vivo in a DSS-induced acute colitis treatment approach, in which colitis was established first and administration of ecto-TMEM219 was then started at day +3. Indeed, treatment with ecto-TMEM219 was associated with an improved DAI score and an increased colon length (Supplemental Figure 5, F–H). Notably, direct inhibition of TMEM219 with a newly generated anti-TMEM219 monoclonal antibody improved colitis phenotype and disease activity (Supplemental Figure 6, A–D). The same effect, to a lesser extent, was also observed with a brand-new anti-IGFBP3 monoclonal antibody (Supplemental Figure 6, E–H). To further test IGFBP3/TMEM219 blockade in a model that better mimics the chronic inflammation of CD, we used the DSS-mediated chronic colitis treatment model, in which colitis was induced with 3 cycles of oral DSS and ecto-TMEM219 was started after the colitis establishment at day +18 (Figure 4H). Inhibition of TMEM219 signaling was associated with improved DAI and histological scores, with rectal bleeding nearly absent, a reduction in some degree of weightloss, and a recovery of colon size (Figure 4, I–K and Supplemental Figure 7, A and B). Importantly, an improvement in crypt architecture and mucosal morphology was also demonstrated in DSS mice receiving ecto-TMEM219 (Figure 4L, upper and middle panels), and further confirmed by an amelioration of the overall endoscopic appearance of the colonic mucosa, with less bleeding, edema, and inflammation (Figure 4, M and N). A reduction in leukocyte infiltration (Figure 4L upper panel and Supplemental Figure 7, C and D) and a replenishment in ISCs and epithelial cells were also observed (Figure 4L lower panel and Supplemental Figure 7, E–H). To this end, ecto-TMEM219 was also able to restore the self-renewal ability of the intestinal crypts in the minigut assay (Supplemental Figure 7, I and J). This regenerative effect was also supported by the measurement of serum IL-22, recently identified as a promoter of mucosal healing and low in DSS-treated mice, which was nearly normalized along with the expression of its receptor, by IGFBP3/TMEM219 inhibition, while no changes were observed in IGF-I levels (Supplemental Figure 7, K–M). This beneficial effect of ecto-TMEM219 was also supported by the downregulation of Caspase 8 (Supplemental Figure 7N). Finally, a colonic transcriptome analysis demonstrated an upregulation of major factors involved in the mucosal stress/damage response and in the ISC-mediated repair with ecto-TMEM219 (Supplemental Figure 7, O–Q). These data confirm that blockade of TMEM219 is associated with an amelioration of acute and chronic colitis, which is linked to an ISC-mediated mucosal healing and repair.

### Pharmacological blockade of IGFBP3/TMEM219 signal improves colitis in a T cell adoptive transfer model.

**Figure 5:**
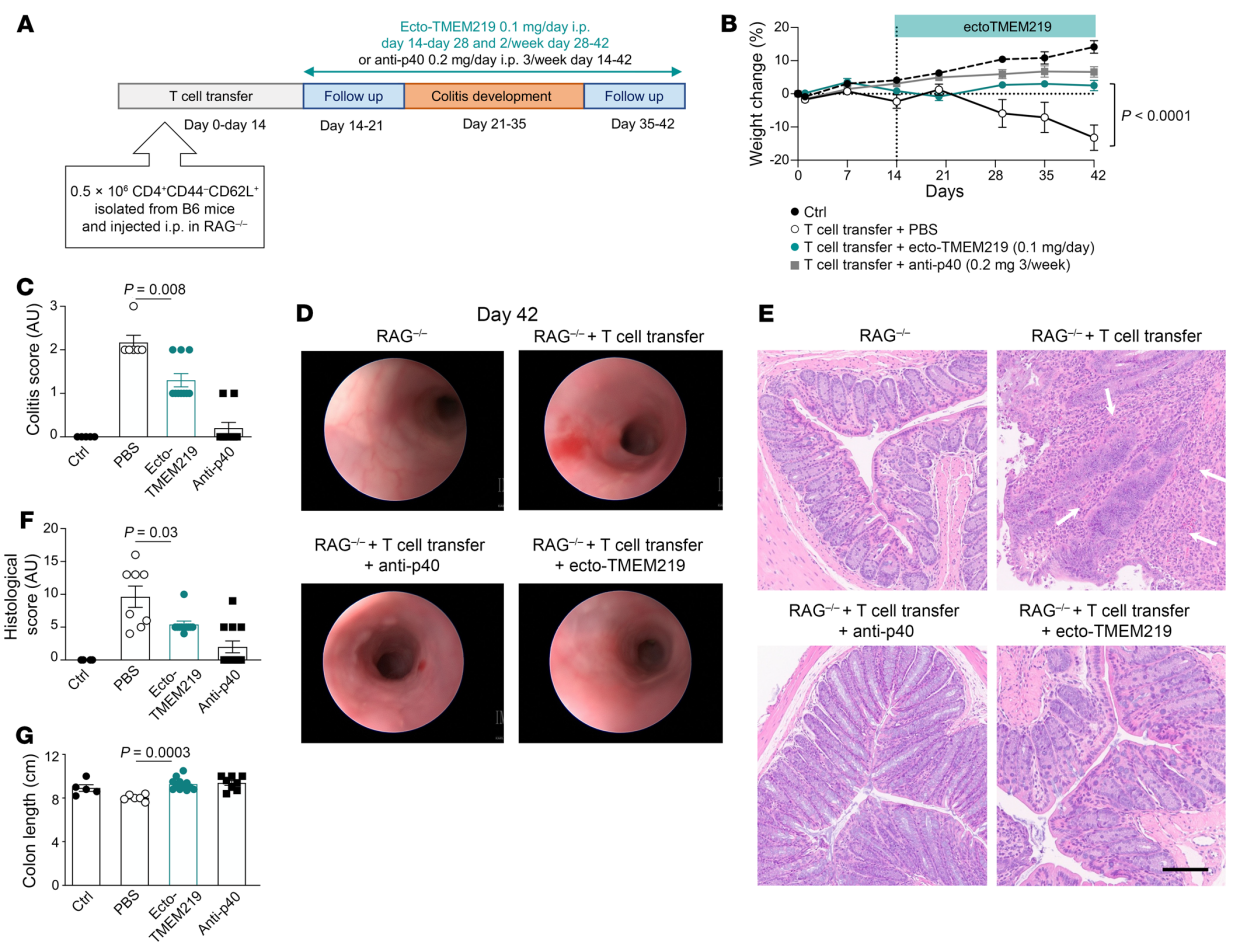
Pharmacological blockade of the IGFBP3/TMEM219 signaling improves colitis in vivo in the T cell transfer model. (**A**). Experimental design of the T cell–mediated acute colitis prevention model, in which mice received T cells at day 0 and developed colitis between days 21 and 35 and were administered treatment with ecto-TMEM219 or with the positive control anti-p40 compound, from day 14 to day 42. (**B**). Percentage weight change in mice subjected to T cell–induced colitis and treated with ecto-TMEM219, anti-p40, or PBS (*n* = 8). (**C** and **D**). Colitis score and representative endoscopic pictures obtained at day 42 in naive mice (RAG^–/–^), in RAG^–/–^ mice receiving T cells plus PBS, T cells plus anti-p40, or ecto-TMEM219 in the T cell transfer model (*n* = 6–10). (**E**). Representative pictures of histological analysis in colon samples of naive mice, mice receiving T cells plus PBS, T cells plus anti-p40, or ecto-TMEM219. Original magnification ×20; scale bar: 100 μm. (**F** and **G**). Histological score and colon length measured at day 42 in naive mice (*n* = 5) and in mice receiving T cells + PBS or T cells + anti-p40 or ecto-TMEM219 in the T cell transfer model (*n* = 8–12). Mean ± SEM. 2-way or 1-way ANOVA followed by Šidák’s post hoc test and 2-sided *t* test, Mann-Whitney *t* test.

To strengthen our findings, we demonstrated in a proof-of-concept study that pharmacological blockade of the IGFBP3/TMEM219 signaling/binding with ecto-TMEM219 successfully improved the signs and symptoms of colitis in a T cell adoptive transfer model, which better parallels the pathogenesis of CD. Briefly, RAG^–/–^ mice were injected with CD44^–^CD62L^+^ T cells isolated from B6 donors and treated after T cell engraftment on day 14 with ecto-TMEM219 or with the reference compound anti-p40 (Figure 5A). First, we observed a reduction in weight loss with ecto-TMEM219, which paralleled that obtained with the reference compound anti-p40 (Figure 5B). An improvement in the colitis severity and stool consistency score was observed in animals treated with ecto-TMEM219 at the endoscopic analysis accounting for a decrease in the colitis score (Figure 5, C and D). An improved histological score, with a reduction in subacute inflammation and in the severity of lesions, such as gland damage/loss, erosions, and epithelial hyperplasia, and an increased colon length measured in ecto-TMEM219-treated mice compared with untreated mice demonstrated that TMEM219 blockade exerts a protective effect in an immunological-mediated colitis model (Figure 5, E–G). Overall, these results indicate that pharmacological blockade of the IGFBP3/TMEM219 signaling/binding with ecto-TMEM219 in vivo ameliorates signs and symptoms of acute colitis and preserves intestinal immune homeostasis.

### Genetic deletion of TMEM219 in ISCs ameliorates acute colitis in vivo.

**Figure 6:**
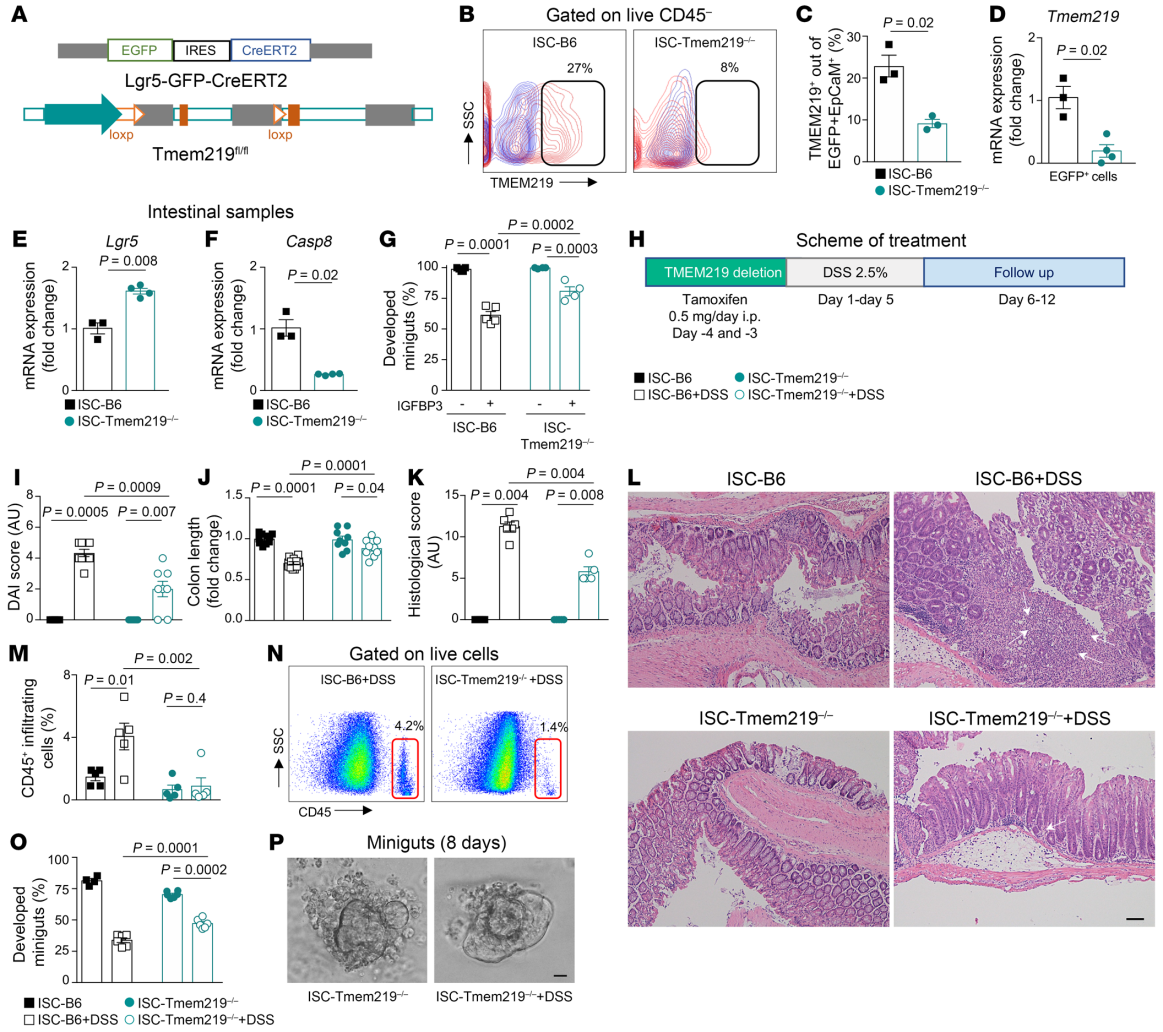
TMEM219 genetic deletion in ISCs ameliorates acute colitis in vivo. (**A**). Genetic approach used to generate the Tmem219^fl/fl^ EGFP-Lgr5^cre^ mouse, namely the ISC-Tmem219^–/–^ mouse. (**B**–**D**). Flow plot and bar graph quantifying TMEM219 protein (**B** and **C**) and mRNA (**D**) expression in EpCam^+^EGFP-LGR5^+^ intestinal cells isolated from the Tmem219^fl/fl^ EGFP-Lgr5^cre^ mouse, in which Tmem219 was deleted through tamoxifen injection (ISC-Tmem219^–/–^, *n* = 3), compared with the ISC-B6 mice, in which Cre was not activated by tamoxifen injection (*n* = 3). (**E** and **F**). Normalized mRNA expression of *Lgr5* and *Casp8* in colons of ISC-Tmem219^–/–^ (*n* = 4) compared with ISC-B6 mice (*n* = 3). (**G**). Bar graphs showing ex vivo–generated 8-day miniguts from crypts of ISC-Tmem219^–/–^ (*n* = 4) and of ISC-B6 controls (*n* = 5) cultured with/without IGFBP3 (50 ng/mL). (**H**). Experimental design of the DSS acute prevention model conducted in ISC-Tmem219^–/–^ mice. (**I**–**K**). DAI score, colon length, and histological score quantified in ISC-Tmem219^–/–^ mice and in the ISC-B6 control (*n* = 8–10) with or without treatment with oral DSS (2.5%) in the prevention acute study model described in **H**. (**L**). H&E staining of colons obtained from mice as described in **I**. Arrows highlight inflammation, infiltrating leukocytes. Original magnification ×10; scale bar: 200 μm. (**M** and **N**). Flow cytometric analysis of infiltrating CD45^+^ cells performed in colon samples of ISC-Tmem219^–/–^ mice and of ISC-B6 control mice with or without treatment with oral DSS, (*n* = 5). (**O** and **P**). Ex vivo–generated 8-day miniguts from crypts of ISC-Tmem219^–/–^ mice and of ISC-B6 control control mice with or without treatment with DSS, (*n* = 6–7). Original magnification ×10; scale bar: 200 μm. Mean ± SEM. 1-way ANOVA followed by Šidák’s post hoc test, 2-sided *t* test.

To further confirm the relevance of the aforementioned axis in ISC death and in the exacerbation of inflammatory colitis, we generated ISC-Tmem219^–/–^ mice by crossing Tmem219^fl/fl^ with EGFP-Lgr5^cre^ mice, in which Tmem219 was conditionally deleted in LGR5^+^ ISCs (Figure 6A). A nearly complete abrogation of TMEM219 expression was demonstrated in LGR5^+^ cells of the ISC-Tmem219^–/–^ mouse by both qRT-PCR and flow cytometry (Figure 6, B–D). An increase in the percentage of LGR5^+^ cells by flow cytometry and downregulation of *Casp8* mRNA in flow-sorted LGR5^+^ cells also demonstrated that ISCs were less prone to apoptosis in the absence of TMEM219 (Supplemental Figure 8, A–C). Indeed, both lower *Casp8* and higher *Lgr5* mRNA expression were confirmed in ISC-Tmem219^–/–^ intestinal samples (Figure 6, E and F). Moreover, the reduced effect of the TMEM219 ligand IGFBP3 on abrogating minigut development in vitro in ISC-Tmem219^–/–^ mice compared with WT animals (ISC-B6) further proved the preservation of regenerative abilities in intestinal crypts and colon length when TMEM219 signaling was abrogated (Figure 6G and Supplemental Figure 8, D and E). Based on these observations, we challenged the ISC-Tmem219^–/–^ mice in the DSS-mediated acute colitis model in both prevention and treatment approaches. In the prevention study, we genetically deleted Tmem219 on days –4 and –3, and orally administered 2.5% DSS for 5 days, thus mimicking the experimental design of the pharmacological blockade of the IGFBP3/TMEM219 signaling (Figure 6H). An improvement in the DAI and histological scores, along with well-preserved colon length, was observed in DSS-treated ISC-Tmem219^–/–^ mice compared with DSS-treated ISC-B6 mice (Figure 6, I–K and Supplemental Figure 8, F and G). Crypt morphology was reestablished and near normalized to that of untreated naive ISC-B6 animals and was also associated with a reduction in leukocyte infiltration in flow cytometric analysis (Figure 6, L–N). The increase in minigut growth ex vivo in DSS-treated ISC-Tmem219^–/–^ mice compared with that in DSS-treated ISC-B6 mice also confirmed that the abrogation of TMEM219 signaling through Tmem219 genetic deletion in ISCs preserved the regenerative potential and promoted the healing of the intestinal mucosa (Figure 6, O and P and Supplemental Figure 8, H and I). To further support the relevance of deleting TMEM219 in ISCs in an immune-mediated enteritis model, we administered a Toll-like receptor–3 agonist, (Poly I:C), which activates immune response, in the ISC-Tmem219^–/–^ and ISC-B6 mice in a preventive approach using a short-term protocol. Weight loss was less evident while leukocyte infiltration was significantly reduced in the ISC-Tmem219^–/–^ compared with the ISC-B6 mice, thereby confirming a beneficial effect of TMEM219 abrogation also in a different enteritis model (Supplemental Figure 8, J–N). We finally tested whether Tmem219 genetic deletion in the ISC-Tmem219^–/–^ mice was effective in a treatment model, in which 2.5% DSS was orally administered for 5 days and Tmem219 genetic deletion was induced after the onset of colitis (Supplemental Figure 9A). Here, abrogation of TMEM219 signaling was associated with an amelioration of the DAI (Supplemental Figure 9, B and C) and of the histological scores compared with those measured in ISC-B6 mice receiving DSS (Supplemental Figure 9, D and E). A relative increase in colon length paralleling that of ISC-Tmem219^–/–^ and WT mice not receiving DSS was also observed (Supplemental Figure 9, F and G). Interestingly, colonic leukocyte infiltration was also reduced, and crypt morphology was nearly restored (Supplemental Figure 9, D, H, and I). This was confirmed by the recovered expression of the ISC markers *EphB2* and *Lgr5* in the intestinal mucosa and was associated with the downregulation of *Casp8* (Supplemental Figure 9, J–L). These findings demonstrate that the tissue-specific genetic deletion of Tmem219 in the intestine preserves regenerative mucosal abilities and halts the onset and progression of colitis.

## Discussion

This study demonstrates the existence of an abnormal TMEM219-mediated ISC death in CD, which exacerbates colitis, limits ISC-dependent mucosal repair, and acts by activating the Caspase-8 signaling. The inability of the intestinal mucosa to self renew plays a significant role in the pathogenesis of CD, and it may be linked to an abnormal activation of TMEM219 deleterious signaling in ISCs. ISC impairment reduces crypt turnover and contributes to increased mucosal permeability, promotes the recruitment of damaging immune cells, and boosts the local inflammatory response (25–27). Therefore, conveying a regenerative signal to the mucosa through the inhibition of cell death, such as that mediated by the TMEM219 pathway, may facilitate the healing of the intestinal mucosa. We are demonstrating here that abnormal cell death is evident in patients with active CD, with low expression of ISC markers and an upregulation of proapoptotic factors, particularly Caspase-8. This further leads to failure of crypt organoids to self renew in active disease, confirming previous observations (28). The failure of self renewal is counteracted in the disease remission and indicates a defect in ISC-mediated tissue repair. It may also suggest that, in CD, ISCs are more fragile and vulnerable to environmental factors and may become dysfunctional and prone to death. In line with this, upregulated expression of TMEM219 was evident in the intestinal samples of patients with active disease and in patients who were nonresponders to several therapies, whereas it was nearly normalized in those patients in the remission phase. The fact that the failure of the intestinal mucosa to self renew was TMEM219 dependent and Caspase-8 mediated was further confirmed by our in vitro and in vivo studies. In vitro, we demonstrated that TMEM219 blockade was associated with the rescue of organoid growth generated from the crypts of patients with active CD. In vivo, abrogation of TMEM219-mediated cell death in acute and chronic DSS–induced colitis using a preventive/curative approach was associated with near normalization of mucosal morphology and ISC regenerative function. Remarkably, in the T cell transfer model, which closely mimics features and development of CD, TMEM219 blockade delayed the disease onset, reduced the colitis severity, and promoted mucosal repair. Finally, genetic inhibition of TMEM219 was associated with an improvement in signs and symptoms of colitis, with reduced infiltration of immune cells. During the development of colitis, cell damage is associated with the release of cell debris and the activation of proinflammatory pathways, which, in turn, promotes the recruitment of immune cells, sustains inflammation, and dysregulates tissue healing (29–32). By halting cell death, blockade of the IGFBP3/TMEM219 signaling prevents the generation of these deleterious signals and indirectly limits the local inflammatory response. The relevance of the IGFBP3/TMEM219 axis in reducing gut inflammation has also been confirmed in the IGFBP3^–/–^ mice that were protected from DSS-mediated colitis (33). Of note, among the 5,152 SNPs identified for TMEM219, none was associated with a disease phenotype up to date. Interestingly, while most compounds developed and tested in in vivo colitis models and in patients with IBD mainly target pathogenic immune cells, proinflammatory factors, and microbe-related processes (34, 35), our study demonstrates that favoring mucosal healing is crucial in ameliorating the disease status of patients with CD, and this points to ISCs as a major driver of success. This appears to be highly clinically relevant when considering that current antiinflammatory and immunosuppressive treatments in IBD, despite significant advances, are still associated with long-term adverse effects and a high rate of disease relapse, with several patients never achieving remission or mucosal healing (36–39). We acknowledge that some limitations exist in our study. First, the DSS-induced colitis in vivo model may not fully account for alterations in the gut microbiota and it may not faithfully replicate the bacteria-dependent origin of CD. Second, to the best of our knowledge, only a few ISC-exclusive markers have been identified, including those recognizing local progenitor cells (e.g., EPHB2 and LGR5) and broad stem cells (e.g., h-TERT, SOX9, and ALDH1A1), but with limited experimental applications (40, 41) and, thus, showing some variability depending on the methods or samples used. Our study also has some major strengths. First, our human study included 4 patient cohorts with different stages of the disease. Second, our in vivo findings in DSS-induced colitis were also confirmed in an immunological-mediated colitis model. In summary, our study demonstrates that a defect in ISCs exists in CD, in which TMEM219 signaling is abnormally activated and a Caspase-8–mediated TMEM219-dependent cell death is evident, leading to the disruption of intestinal repair from damage. It also establishes that the blockade of the TMEM219 detrimental signaling may rescue the ability of the mucosa to self renew and heal. This approach appears highly translational based on the following major observations: (a) the IGFBP3/TMEM219 biology is highly preserved across species, including humans, thus reinforcing the relevance of our preclinical data; (b) in vitro and in vivo blockade of the axis consistently showed positive results; (c) the IGFBP3/TMEM219 binding or its inhibition with ecto-TMEM219 did not show any interference with peripheral IGF-I (Supplemental Figure 10, A–C); (d) the treatment of IBD is still dominated by immune system modulation and studies focused on epithelial cell regeneration are urgently needed; (e) based on a more restrictive view on TMEM219 expression in human tissues and its upregulation only in active disease, lack of unwanted side effects should be expected, thereby making TMEM219 targeting on ISCs very much feasible.

## Methods

A detailed description of the methods is provided in the Supplemental Methods.

### Sex as a biological variant

We included both male and female sexes in the human and mouse studies. Sex was not considered as a biological variable in the studies.

### Human studies

Samples (tissue and blood) were obtained from 112 patients with CD (39 with active disease, 34 patients who were responding to medical therapy and in remission phase, 39 patients who did not respond to therapy, based on the clinical and endoscopic scores of CD Index of Severity, Simple Endoscopic Score for CD, and Rutgeerts score for postsurgery disease recurrence), and 39 participants who were healthy controls and who were without a diagnosis of CD. All participants provided informed consent (Supplemental Table 1). Intestinal samples of patients with CD were obtained by surgery (full thickness section) or endoscopy (pinch biopsy) during routine clinical practice from different patient cohorts. In patients with active disease, samples were obtained from the marginal area, sampled at 5–10 cm from the inflammatory lesion to avoid the presence of inflammation, and from the inflamed area, which was excised within 5 cm from the lesion. Patients who were not responding to conventional first-line or second-line therapies (e.g., anti-TNF-a, corticosteroids, and immunomodulators) were considered nonresponders. Patients undergoing colonoscopy/surgery as a routine procedure for gastrointestinal symptoms of other origins and/or for colorectal cancer screening/resection who had no history of CD were included as controls. Intestinal samples were processed as described in Supplemental Methods and analyzed to assess ISC-based transcriptome profile, apoptotic signature, TMEM219 expression, cell apoptosis and death. To this end, flow cytometry analysis, ELISA, immunostaining, large crypts organoids generation and culturing and mRNA analysis have been employed. To understand the relevance of the TMEM219-related cell death mechanism in CD and the downstream signaling involved, a Caspase-8 interactome (42) and a phosphoproteome profile were delineated (Supplemental Tables 5 and 6). For mechanistic in vitro studies conducted on organoids and on intestinal cells, recombinant human IGFBP3 (50 ng/mL, 8874-B3, R&D Systems), and ecto-TMEM219 cloned into the TMEM219 extracellular domain (130 ng/mL, Genscript) were used (22).

#### Intestinal cell lines.

The CaCo2 human cell line was purchased from ATCC (HTB-37) and originally derived from human colon adenocarcinoma. Cells were cultured in RPMI 1640 with 10 % FBS, 1 % NEAA, 50 μM thioglycerol, and 1 × Penicillin-Streptomycin (all from ATCC).

#### Cell death analysis and downstream signaling.

To assess apoptosis/cell death in isolated human crypts and CaCo2 cell line, we employed a photometric enzyme immunoassay (11544675001, Roche Diagnostics GmbH), which quantifies in vitro the histone-associated DNA fragments after inducing cell stress in cell cytoplasmic lysates and cell supernatants. Apoptosis was analyzed using flow cytometry in human crypts isolated from intestinal specimens, including marginal and inflamed samples from patients with CD, and stained with propidium iodide (PI), Annexin V FITC, CD45, and EPHB2, all from BD Biosciences (see *Flow cytometry* in the Supplemental Methods). Cleaved Caspase 8 and phosphorylated-AKT were assessed using ELISA (MBS766157, MyBiosource and KHO0111, Invitrogen) in human crypts, patient-derived organoids, and CaCo2 cells cultured with/without IGFBP3 (50 ng/mL, 8874-B3, R&D Systems), with or without ecto-TMEM219 (130 ng/mL, Genscript) (22).

#### TMEM219 expression and flow cytometry studies.

TMEM219 protein expression was analyzed in the lysates of purified human crypts using ELISA (MBS9341285, MyBioSource ELISA) according to the manufacturer’s instructions. Single cells obtained from purified crypts were stained with propidium iodide (10 μg/mL) to exclude dead cells and with V450 anti-human CD45 (clone HI30, 560368, BD Biosciences) or with BD Horizon BV421 anti-Human CD45 (clone HI30, 563880, BD Biosciences) to exclude infiltrating immune cells. Primary human anti-TMEM219 (courtesy provided by Yumab GmbH, Braunschweig, Germany) was used to detect TMEM219 expression in combination with a secondary PE goat anti-human IgG (12-499-82, Thermo Fisher Scientific). Flow cytometry analysis was performed using a BD FACS Celesta flow cytometry system (BD Biosciences) and analyzed using FlowJo software (Version 10, Tree Star).

### Animal studies

#### DSS colitis model.

In the acute colitis model, 8-week-old B6 mice received 2.5% of DSS (45 kD; TDB Consultancy AB, Uppsala, Sweden, Batch number DB001-42; 42867, Sigma-Aldrich) in drinking water for 5 days, followed by 1 week of regular water (43). Ecto-TMEM219 was administered (0.1 mg/day, i.p.) from day –3 to day +12 in the prevention protocol and from day +3 to day +12 in the treatment protocol. In the chronic colitis model, B6 mice received 3 oral cycles of 2% DSS (40 kDa; MP Biomedicals), followed by 1 week of regular drinking water. Ecto-TMEM219 was administered 0.1/mg/mouse daily i.p., days 18–32, then twice per week. At day 42, the animals were subjected to endoscopy and euthanized (44).

#### T cell adoptive transfer model.

Colitis was induced on day 0 in RAG2^–/–^ mice (Taconic Biosciences) by i.p. injection of 0.5 × 10^6^ CD44^–^/CD62L^+^ T cells isolated and purified from spleen of C57Bl/6 donors through CD4-negative selection kit (130-104-454, Miltenyi) and enriched in naive T cells (CD44^–^ /CD62L^+^) by using the naive T cell isolation kit (130-104-453, Miltenyi). Animals received vehicle (PBS) daily (days 13–28), ecto-TMEM219 (0.1 mg/mouse daily days 14–28, every 3 days, days 31–42) or with anti-p40 (10 mg/Kg, every 3 days, days 13–42, positive control). At day 42, colitis severity, histopathology, and colon length were analyzed. Disease activity was evaluated as following: 0, normal; 1, loss of vascularity; 2, loss of vascularity and friability; 3, friability and erosions; and 4, ulcerations and bleeding.

#### Tmem219^fl/fl^Lgr5^cre^ model.

In order to demonstrate the effect of Tmem219 genetic ablation on ISCs, ISC-Tmem219^–/–^ mice generated by breeding Tmem219^fl/fl^ mice (21) with Lgr5^cre^ mice (B6.129P2-Lgr5tm1(cre/ERT2)Cle/J, Jackson Laboratories, 008875) (45) were injected with tamoxifen (20 mg/mL, T5648, Sigma Aldrich 100 μL, i.p., day –4 and –3) to induce Cre-mediated deletion of Tmem219 on LGR5^+^ cells. ISC-Tmem219^–/–^ mice in which tamoxifen was not injected were used as controls. For colitis induction, mice received 2.5% DSS in drinking water for 5 days and were sacrificed at day +12. In the treatment study, tamoxifen was injected on days +7 and +8.

### Statistics

Continuous variables are presented as means with standard errors, and categorical variables are presented as proportions. Independent sample 2-sided, 2-tailed *t* tests (Student’s *t* test for normally distributed data or Mann-Whitney U test for data without normal distribution) were used to compare continuous variables, while Fisher’s test was used for categorical variables. For multiple comparisons, 1-way or 2-way ANOVA followed by Tukey/Šidák’s post hoc tests were used, while Kruskal-Wallis analysis was used for multiple nonparametric data. 2-tailed *P* values of less than 0.05 were considered statistically significant. All analyses were conducted by using GraphPad Prism V7/V9.

### Study approval

All human studies were conducted after obtaining appropriate Institutional Review Board approval (Stem Cells IBD n. 2017/ST/277, Ethic Committee Milano Area 1). All studies were conducted in compliance with the relevant ethical regulations for studies involving human participants. All participants provided written informed consent. Animal studies were approved by the local review board (Nord-Pas-deCalais CEEA 75, Lille, France; n. 352012 and 19-2009R, APAFIS#7542-20 17030609233680) and by the Italian Ministry of Health (98/2022-PR and 1144/2020-PR).

### Data availability

The data supporting the findings of this study are available in the Supporting Data Values file. The raw data or noncommercial materials used in this study are available from the corresponding author upon reasonable request. Any data or materials that can be shared are released via a Material Transfer Agreement.

## Author contributions

FD designed the study, analyzed the data, and wrote the paper. GA analyzed the data. EA, AM, AP, MBN, VU, M Zocchi, and CL, IP, and MEL performed the experiments and analyzed the data. AA, AJS, VM, M Zangarini, SP, FC, CN, and MN M Nardini collected the preclinical samples and analyzed the data. DC, SLR, and M Nebuloni performed immunostaining and histological analyses. GMS and SA coordinated human sample collection. BEE, JY, MV, GM, FF, SD, GZ, and SA coordinated and designed the research and edited the paper. PF conceived the idea, designed the study, and wrote and edited the paper.

## Supplementary Material

Supplemental data

Supplemental table 5

Supporting data values

## Version 1 05/15/2025

Electronic publication
