# Supplementary material for: TMEM219 signaling promotes intestinal stem cell death and exacerbates colitis
Source: J Clin Invest. 2025 May 15;135(10):e185783. doi: 10.1172/JCI185783 (PMC12077909; doi:10.1172/JCI185783)
Supplement: Supplemental data [file jci-135-185783-s167.pdf]

SUPPLEMENTARY INFORMATION

TMEM219 signaling promotes intestinal stem cell death and exacerbates colitis

Francesca D'Addio[1,2,#], Giovanni Amabile[3#], Emma Assi[1#], Anna Maestroni[1], Adriana Petrazzuolo[1], Cristian Loretelli[1], Ahmed Abdelasalam[1], Moufida Ben Nasr[1,4], Ida Pastore[2], Maria Elena Lunati[2], Vera Usuelli[1], Monica Zocchi[1], Andy Joe Seelam[1], Domenico Corradi[5], Stefano La Rosa[6], Virna Marin[3], Monique Zangarini[3], Marta Nardini[3], Stefano Porzio[3], Filippo Canducci[3], Claudia Nardini[3], Basset El Essawy[7,8], Manuela Nebuloni[9], Jun Yang[10], Massimo Venturini[11], Giovanni Maconi[12], Franco Folli[13], Silvio Danese[14], Gianvincenzo Zuccotti[15], Gianluca M. Sampietro[16], Sandro Ardizzone[12] and Paolo Fiorina[1,2,4*]

[1]International Center for T1D, Pediatric Clinical Research Center Romeo ed Enrica Invernizzi, Department of Biomedical and Clinical Sciences, Università degli Studi di Milano, Milan, Italy; [2]Division of Endocrinology, ASST Fatebenefratelli-Sacco, Milan, Italy; [3]Enthera, Milan, Italy; [4]Boston Children's Hospital and Transplantation Research Center, Brigham and Women's Hospital, Harvard Medical School, Boston, MA, USA; [5]Department of Medicine and Surgery, Unit of Pathology, University of Parma, Parma, Italy; [6] Department of Medicine and Surgery, Università degli Studi dell'Insubria, Varese, Italy;[7]Nephrology Division, Brigham and Women's Hospital, Harvard Medical School, Boston, MA, USA; [8]Medicine, Al-Azhar University, Cairo, Egypt; [9]Pathology Unit, ASST-Fatebenefratelli Sacco and Department of Biomedical and Clinical Sciences, Università degli Studi di Milano, Milan, Italy; [10]Institute of Organ Transplantation, Tongji Hospital and Medical College, Huazhong University of Science and Technology, Wuhan, China; [11]Diagnostic and Interventional Radiology Department, Circolo Hospital, ASST Sette Laghi and School of Medicine and Surgery, Insubria University, Varese, Italy; [12]Gastrointestinal Unit, ASST-Fatebenefratelli Sacco and Department of Biomedical and Clinical Sciences, Università degli Studi di Milano, Milan, Italy; [13]Endocrinology and Metabolism, Department of Health Science, Università di Milano, Diabetic and Metabolic Diseases Unit-ASST Santi Paolo e Carlo, Milan, Italy; [14]Gastroenterology and Endoscopy, IRCCS Ospedale San Raffaele and Medicine and Surgery Department, Vita-Salute San Raffaele University, Milan, Italy; [15]Pediatric Clinical Research Center Romeo ed Enrica Invernizzi-Università di Milano and Buzzi Children's Hospital, Milan, Italy; [16]Division of General and HBP Surgery, Rho Memorial Hospital, ASST Rhodense, Milano, Italy; [*]Corresponding Author; [#]Authors share co-first authorship.

TABLE OF CONTENTS:

SUPPLEMENTARY METHODS

SUPPLEMENTARY TABLES 1-6

SUPPLEMENTARY FIGURES 1-10

SUPPLEMENTARY METHODS

Human studies

Patients and study design

Samples (blood, tissue) were obtained from 112 patients with Crohn’s disease (39 with active disease, 34 patients who were responding to medical therapy and in remission phase and 39 patients who did not respond to therapy, based on the clinical and endoscopic scores of Crohn's Disease Index of Severity, Simple Endoscopic Score for Crohn's Disease, and Rutgeerts score for post-surgery disease recurrence) and 39 healthy control participants without a diagnosis of Crohn’s disease who provided informed consent (Supplementary Table 1). Intestinal tissues of patients with active disease were samples from the marginal and from the inflamed area obtained by surgery or endoscopy during routine clinical practice. The marginal area was sampled at 5-10 cm from the inflammatory lesion to avoid the presence of inflammation, while the inflamed sample was excised within 5 cm from the lesion. Patients undergoing colonoscopy/surgery as a routine procedure for gastrointestinal symptoms of other origins and/or for colorectal cancer screening/resection who had no history of Crohn’s disease were included as controls. This study was conducted after obtaining appropriate Institutional Review Board approval (Stem Cells IBD n. 2017/ST/277, Ethic Committee Milano Area 1). All studies were conducted in compliance with the relevant ethical regulations for studies involving human subjects.

In vitro studies

Tissue specimens

Tissue specimens of patients with Crohn’s disease were selected and sampled by a specilized gastroenterologist as following: “marginal” tissue was obtained from noninflamed regions 5-10 cm distant from the pathologic lesion and defined as mucosa areas without macroscopic/endoscopic

signs of inflammation (e.g., discoloration, hemorrhagic appearance, edema, ulceration, or mucinous/fibrinous coating); “inflamed” tissue was obtained from inflamed areas within 5 cm from the lesion and with detectable macroscpic/endocopic signs of inflammation (1). Samples collected during colorectal surgery were selected following the same criteria for patients with Crohn’s disease and absence of inflammation was validated by an expert pathologist. Tissue specimens from controls were obtained from patients undergoing abdominal surgery for colon cancer or polyposis, which had non-involved left side colon removed, as part of the surgery.

Human crypts isolation

Crypts were extracted from mucosa and sub-mucosa of intestinal samples obtained from patients with Crohn’s disease or from control subjects. After incubation with a mixture of antibiotics, tissue was minced into small pieces and incubated with 10 mM Dithiothreitol (DTT) (Sigma) in PBS. Sample was then transferred to 8 mM EDTA in PBS and incubated for 30 minutes at 37°C. After this step, vigorous shaking of the sample yielded supernatants enriched in colonic crypts, which were then dissociated and used for flow cytometry studies, qRT-PCR, expression studies by ELISA and *in vitro* generation of crypts organoids, namely mini-guts. The procedure does not allow to exclude presence of intraepithelial lymphocytes, although in a very limited percentage (2-5%).

Transcriptome profiling

Total RNA was extracted from purified intestinal crypts using an RNeasy Mini Kit (Qiagen, Valencia, CA, USA) with on-column DNase I digestion. Total RNA from each sample (3 μg) was reverse-transcribed using an RT2 First Strand kit (C-03; Qiagen). To delineate the transcriptome profile of stem cell related markers a customized version of the Human Stem Cell RT2 Profiler PCR Arrays (Qiagen), which included the analysis of ISC markers(2) (full list of genes analyzed: AXIN1, CCNA2, CCND1, CCND2, CCNE1, FGF1, FGF2, MYC, NOTCH2, KAT2A, HDAC2,

KAT8, TERT, NOTCH1, NUMB, HSPA9, NEUROG2, SOX1, SOX2, BMP1, JAG1, ALDH1A1, FOXA2, WNT1, *AXIN2, OLFM4, BMI1, RNF43, CDCA7, SLC12A2, CDK6, SOX9, DKC1, ZNRF3, ETS2, EPHB2, FAM84A, LGR5, GPX2*) was used while the apoptosis transcriptome analysis was conducted by using the Human Apoptosis PCR Arrays (PAHS-012Z, Qiagen). Statistical analysis was used to compare gene expression across all cell populations for each patient using one-way ANOVA, followed by a Bonferroni post-hoc test for multiple comparisons between the population of interest and all other populations. The analysis was performed using RT^2 Profiler PCR Array Data Analysis software (Qiagen).

LGR5 in situ hybridization (ISH)

ISH for LGR5 expression was performed using the RNAscope 2.0 High Definition (Red, catalog number 310036) assay on 3-5-µm-thick histological sections according to the manufacturer's instructions (Advanced Cell Diagnostics, Hayward, CA) and as already described (3). Slides were incubated with the probes for 2 hours at 40°C, followed by successive incubations with Amp1 to 6 reagents. Staining was visualized with 3,3′-diaminobenzine (DAB) for 10 minutes, then lightly counterstained with Gill's haemotoxylin. RNAscope probe used was LGR5 (NM_003667.2, region 560–1589, catalog number 311021). LGR5 expression at the crypt base was quantified according to the five-grade scoring system recommended by the manufacturer (0 = No staining or less than 1 dot to every 10 cells (40× magnification), 1 = 1–3 dots/cell (visible at 20–40× magnification), 2 = 4–10 dots/cell, very few dot clusters (visible at 20–40× magnification), 3 = > 10 dots/cell, less than 10% positive cells have dot clusters (visible at 20× magnification), 4 = > 10 dots/cell. More than 10% positive cells have dot clusters (visible at 20× magnification)). At least 3 patients per group, with an average of 15 crypts scored per sample group were analyzed and scored.

TMEM219 expression studies

TMEM219 protein expression was analyzed in the lysates of purified human crypts using ELISA (MBS9341285, MyBioSource ELISA, San Diego, CA, USA) according to the manufacturer's instructions and analyzed as a fold-change vs. controls. IGFBP3 expression in intestinal samples was analyzed by ELISA (RAB0235, Merck) following the manufacturer's instructions.

Cell death analysis and downstream signaling

To assess apoptosis/cell death in isolated human crypts and CaCo2 cell line, we employed a photometric enzyme immunoassay (11544675001, Roche Diagnostics GmbH, Mannheim, Germany), which quantifies *in vitro* the histone-associated DNA fragments after inducing cell stress in cell cytoplasmic lysates and cell supernatants. Apoptosis was analyzed using flow cytometry in human crypts isolated from intestinal specimens, including marginal and inflamed samples from patients with Crohn's disease, and stained with propidium iodide (PI), Annexin V FITC, CD45, and EPHB2, all from BD Biosciences (see *Flow cytometry*). Cleaved Caspase 8 and phosphorylated-AKT were assessed using ELISA (MBS766157, MyBiosource, San Diego, CA, USA and KHO0111, Invitrogen, Waltham, USA) in human crypts, patient-derived organoids and CaCo2 cells cultured with/without IGFBP3 (50 ng/mL, 8874-B3, R&D Systems), with or without ecto-TMEM219 (130 ng/mL, Genscript) for 72 h.

Caspase 8 interactome analysis

The gene function prediction web-based interface Genemania (4, 5) was used to generate a molecular function-based weighted network for Caspase 8, which was selected using Gene Ontology and focused on physical interactors and co-expression markers. The top 100 genes interacting with Caspase 8 are reported in Supplementary Table 4. Interactome analysis was further confirmed using other web-based prediction tools, such as IntAct-EMBL-EMI and Protein Interaction Network Analysis 3.0. Among the 100 genes interacting with Caspase 8, we selected

those expressed on the cell membrane, leading to the exclusion of 92 factors that mainly participate as intracellular inducers and adaptors of Caspase 8-mediated cell death. We also excluded those receptors primarily linked to the lymphoid compartment (n=3) and those associated with other inflammatory-mediated signaling pathways (i.e., TNF-alpha), as this may represent a confounding factor in assessing the relevance of caspase 8-mediated apoptosis in intestinal stem cells. This then left one factor, namely, TMEM219. The MIscore of interaction between Caspase 8 and the top 8 proteins showing an *in vitro* validated physical association in human species, including TMEM219, was calculated by IntAct-EMBL-EMI.

Phospho-proteomic analysis

A phospho-proteomic array (PEX100-UMCL, Tebubio, Italy) was used to measure changes in phosphorylation status at specific sites in proteins extracted from CaCo2 cells cultured with or without IGFBP3 (50 ng/mL) and in the presence/absence of ecto-TMEM219 (130 ng/mL). A total of 1318 antibodies were tested and covalently immobilized (six replicates) on a glass surface coated with a unique 3D polymer ensuring high binding efficiency and specificity. Each array included well-characterized and relevant antibodies, as well as positive and negative controls. Fluorescence was measured using a microarray scanner. The median signal intensity was extracted from the array image of each spot on the array. For each antibody, using the median signal intensity (F532 Median), the average signal intensity of the replicate spots and the coefficient of variation for the replicate spots for each antibody were calculated. For normalization, within each array slide, the median value of the average signal intensity for all antibodies on the array was determined and calculated as well as the fold change between the control and treated samples (increase in expression ratio ≥ 2 and decrease ratio ≥ 0.5). Using the average signal intensity of replicate spots on the array for each pair of phospho-antibodies and non-phospho-antibodies, the signal ratio of

phospho-protein to non-phospho-protein and the fold change were also determined (increase in expression ratio ≥ 2 and decrease ratio ≥ 0.5). All the detected phosphorylated proteins are listed in Supplementary Table 5.

Flow cytometry

Single cells obtained from purified crypts were stained with propidium iodide (10 µg/mL) to exclude dead cells and with V450 anti-human CD45 (clone HI30, 560368, BD Biosciences, San Jose, CA, USA) or with BD Horizon™ BV421 anti-Human CD45 (clone HI30, 563880, BD Biosciences, San Jose, CA, USA) to exclude infiltrating immune cells. BV711 mouse anti-human EphB2 (Clone 2H9, 743766, BD Biosciences) and OptiBuild™ BV421 Rat Anti-Human Lgr5 (Clone 8F2, 752791, BD Biosciences) were used to assess the expression of the ISC markers EPHB2 and LGR5. Primary human anti-TMEM219 (courtesy provided by Yumab GmbH, Braunschweig, Germany) was used to detect TMEM219 expression in combination with a secondary PE goat anti-human IgG (12-499-82, Thermofisher). For apoptosis analysis, FITC Annexin V (560931, BD Biosciences) positive staining was analyzed in $CD45^{-}PI^{-}EphB2^{+}$ cells, and the fold positivity was measured in Crohn's disease-derived crypts as compared to those from control patients. Flow cytometry analysis was performed using a BD FACS Celesta flow cytometry system (BD Biosciences) and analyzed using FlowJo software (Version 6 and Version 10, Tree Star, Ashland, OR, USA).

Recombinant proteins and interventional studies

Recombinant human IGFBP3 expressed in S9-baculovirus with a molecular weight of 30 kDa and a purity > 95% (50 ng/mL, 8874-B3, R&D Systems), recombinant human IGF-I (50, 100, 200, 500 ng/ml, 8 kDa, I3769, Merck) and ecto-TMEM219 cloned into the TMEM219 extracellular domain with a molecular weight of 18 kDa and a purity > 90% (130 ng/mL, Genscript, 1:1 molar ratio vs.

IGFBP3) (6) were added to cell/mini-gut cultures on day +1. Ecto-TMEM219 was also administered intraperitoneally *in vivo* (i.p.) at a dose of 0.1 mg/mouse/day for 15 days to 10-week-old C57BL/6J mice that were also receiving 2.5% DSS orally for 5 days in a preventive and treatment setting. A dose of 0.1 mg/mose/day for 18 days and then twice a week until day 42 was used in mice receiving oral 2.0% DSS in three repeated 5 days cycles. To generate intestinal stem cell–specific Tmem219$^{-/-}$ mice, mice harboring exon 4 of the Tmem219 gene flanked by loxP sites (Tmem219$^{flox/flox}$) were crossed with mice expressing a CreERT2 fusion protein in the Lgr5 promoter (B6.129P2-Lgr5tm1(cre/ERT2)Cle/J) from Jackson Laboratories (Bar Harbor, ME, USA). Mice were injected with tamoxifen (20 mg/mL, 100 μL; T5648 Sigma-Aldrich) on two consecutive days to activate the Cre recombinase deletion of the floxed sequences.

Intestinal Cell lines

The CaCo2 human cell line was purchased from ATCC (HTB-37) and originally derived from human colon adenocarcinoma. Cells were cultured for 72 hours with/without the recombinant proteins reported in the *Recombinant proteins and interventional studies* section. The following Caspase inhibitors Pan Caspase inhibitor (Z-VAD-FMK, #FMK001, R&D Systems), Caspase 1 inhibitor (#400010-1mg, Sigma-Aldrich), Caspase-3 inhibitor (Z-DEVD-FMK, #FMK004, R&D Systems), Caspase 7 inhibitor (Caspase 7 blocking peptide, #SBP3467, Sigma-Aldrich), Caspase-8 inhibitor (Z-IETD-FMK, #FMK007, R&D Systems), Caspase-9 inhibitor (Z-LEHD-FMK, #FMK008, R&D Systems) were all tested at a concentration of 20 μM in CaCo2 human cell line with/without IGFBP3 (50 ng/ml) and cell death was analyzed after 72 hours. Cells were also cultured with/without IGFBP3 (50 ng/ml) and activation of Human Caspase 1, 3, 7, 8 and 9 was quantified by ELISA (#MBS7254681-96, Human Cleaved-Caspase-1, MyBiosource; #ab22065, Human-Cleaved-Caspase-3 Abcam; #ab275900, Human-Cleaved-Caspase-7, Abcam;

#HUFI04740 Human Cleaved Caspase-9, AssayGenie). Cells were also transfected with 37,5 ng of small interfering RNA (siRNA; Flexitube siRNA SI04381013, Qiagen) in culture medium and with 6 μl HiPerFect Transfection Reagent (Qiagen) were incubated at room temperature to allow for the formation of transfection complexes. Cells further were incubated with these transfection complexes under their normal growth conditions for 6 h and analyzed for cleaved Caspase 8 quantification at 72 h. Finally, cells were also cultured upon the following stimuli: glucose 35 mM, IGFBP3 (50 ng/ml), H2O2 (400 μM), Thapsigargin (3 μM, T9033, Merck), cytokines (IL-1b, 20 ng/ml, #201-LB, IFN-g, 10 ng/ml #285-IF, and IL-6, 20 ng/ml #206-IL, all from R&D Systems, TNF-a 10 ng/ml #10291, BioTechne) and TMEM219 expression and cell death were evaluated.

Mini-guts generation

Crypts (200-300) obtained as described above were mixed with matrigel and plated on pre-warmed culture dishes. After solidification, crypts were overlaid with complete crypt culture medium consisting of Wnt3a-conditioned medium and Advanced DMEM/F12 (Life Technologies) in a 50:50 ratio, supplemented with Glutamax, 10 mM HEPES, N-2 [1×], B-27 without retinoic acid [1×], 10 mM Nicotinamide, 1 mM N-Acetyl-L-cysteine, 50 ng/ml human EGF (Life Technologies), 1 μg/ml RSPO1 (Sino Biological), 100 ng/ml human Noggin (Peprotech, London, UK), 1 μg/ml Gastrin (Sigma-Aldrich), 500 nM LY2157299 (Axon MedChem, Groningen, The Netherlands), 10 μM SB202190 (Sigma-Aldrich) and 0.01 μM PGE2 (Sigma-Aldrich). Medium was replaced every ~~3~~ 2 days. Developed mini-guts with at least 1 crypt domain were counted, and their percentage was calculated based on the mini-guts developed at day +1 and +8. Images of mini-guts were taken by using an inverted microscopy Leica DH/RB and acquired with Axio

Vision AC Release 4.3. Pictures reported in figures represent mini-guts at day 8, 10X magnification.

Mini-guts culturing conditions

Human and murine mini-guts were cultured with/without IGFBP3 (50 ng/ml, 8874-B3, R&D Systems), with/without ecto-TMEM219 (130 ng/ml in a 1:1 molar ratio) and the percentage of developed mini-guts based on the presence of at least one crypt domain detected at day 8 was measured. Medium was changed every 48 hours and IGFBP3/ecto-TMEM219 were newly added. To culture purified crypts with culturing medium containing human serum in place of regular FBS, L-Wnt3 cells were grown in 10% human serum to generate a conditioned medium added 50:50 to Advanced DMEM/F12 medium in order to generate mini-guts *in vitro*. To parallel the Crohn's disease microenvironment, human serum obtained from patients with active disease, or from responder patients in remission phase or not responding to conventional therapy (n=5/group) was pooled and added in place of regular FBS at a concentration of 10% to mini-guts, which were cultured for 8 days as described above and development of organoids was compared with that measured in the presence of pooled serum of controls (n=5) at a concentration of 10% (in place of regular FBS) or regular culturing medium (with 10% FBS). After 8 days, human and murine mini-guts were collected, and expression of ISC markers and of *Casp8* (Life Technologies) were examined by qRT-PCR. Cleaved Caspase 8 and cell death were assessed by ELISA and data were normalized per total protein quantification.

qRT-PCR and transcriptome analysis

RNA from purified crypts was extracted using TRIzol Reagent (Invitrogen, Carlsbad, CA, USA), reverse-transcribed using High Capacity cDNA Reverse Transcription kit (Life Technologies) and qRT-PCR analysis was performed using TaqMan assays (Life Technologies) according to the

manufacturer's instructions. Quantitative reverse transcriptase polymerase chain reaction (qRT-PCR) data were normalized for the expression of *ACTB* (human samples) or *Hprt* (murine samples), and ΔΔCt (fold change) was calculated. For comparison between two groups, a student's *t* test was employed. In Supplementary Table 6 the main characteristics of primers used for human and murine genes are reported. For transcriptome analysis conducted in murine colon samples we used the mouse Chron's disease RT2 Profiler PCR Arrays (PAMM-169Z, Qiagen) implemented with a customized array which included the following relevant genes: *Casp8, Lgr5, EphB2, Mki67, Aldh1a1*. One-way ANOVA, followed by a Bonferroni post-hoc test for multiple comparisons between the population of interest and all other populations was used for statistical analysis through the RT^2 Profiler PCR Array Data Analysis software (Qiagen). Top enriched pathways were identified based on the top 30 genes up/downregulated in colon obtained from chronic DSS+PBS treated mice as compared to those treated with chronic DSS+ecto-TMEM219. The log10 fold enrichment was calculated and the top 8 pathways were graphed by using the ShinyGO 0.77 software.

Luminex and serum analysis

Levels of cytokines were assessed in human plasma samples in duplicates by using the Bio-Plex Pro human cytokine 17-plex panel (M5000031YV, Bio-Rad Laboratories, Milan, Italy) and read using the Bio-Plex 200 System (Bio-Rad Laboratories). Data processing was performed using Bio-Plex manager (software version 6.1.1) to analyze mean values. The ProQuantum™ mouse IL22 immunoassay (MAN0017048, ThermoFisher), which detects the antibody-antigen binding through qPCR technology, was used to quantify IL-22 in mouse serum.

***In vitro* binding studies**

Binding between IGFBP3 and the extracellular portion of TMEM219 (Ecto-TMEM219) was tested by coating the ELISA plate with IGF-I (2 mg/ml) and by adding rhIGFBP3 and ecto-TMEM219 at increasing concentrations. Absorbance of either ecto-TMEM219 with anti-His HRP (GeneTex) or IGFBP3 with anti-IGFBP3 HRP were analysed by ELISA. Binding between ecto-TMEM219 and IGF-I was tested by measuring the absorbance of IGF-I with anti-IGF-I HRP antibody (Invitrogen) through ELISA. Negative control was PBS coated plate.

Animal studies

Eight-week-old C57BL/6J (B6) mice were purchased from Charles River Laboratories (#632, SAS, France), while Lgr5^{cre} (B6.129P2-Lgr5tm1(cre/ERT2)Cle/J) mice were obtained from Jackson Laboratories (008875, Bar Harbor, ME) (7). Tmem219$^{flox/flox}$ mice were generated in collaboration with Applied StemCell (Milpitas, CA, USA) and housed at Charles River Laboratories (Calco, Italy). Tmem219$^{flox/flox}$Lgr5^{cre} mice (ISC-TMEM219$^{-/-}$) were obtained by breeding homozygous Tmem219$^{flox/flox}$ mice with heterozygous Lgr5^{cre} mice and were housed at Charles River Laboratories (Calco, Italy). All mice were cared for and used in accordance with the Italian law on animal care N° 116/1992 and European Communities Council Directive EEC/609/86. Three to five animals were housed per cage and had free access to standard mouse chow and tap water. The animals' room conditions were as follows: temperature, 20 ± 5°C; humidity, 50 ± 20%; with 12h/12h as light/dark cycle and ventilation of 12 cycles/hour of filtered non-recycled air.

DSS colitis model

In the acute colitis model, 8 week-old B6 mice received 2.5% of DSS (45kD; TDB Consultancy AB, Uppsala, Sweden, Batch number DB001-42; 42867, Sigma-Aldrich, St Louis, MO, USA) in their drinking water for 5 days, followed by a regimen of 7 days of regular water (8). In the prevention protocol, ecto-TMEM219 was administered from day -3 to day 12 (0.1 mg/day i.p. and

PBS was used as the vehicle control). In the treatment model, ecto-TMEM219 was administered at the same dose starting at day 3 until day 12. Mice were sacrificed on day 12 for mechanistic studies. Animal studies were approved by the local/national review board (Nord-Pas-de-Calais CEEA 75, Lille, France; n. 352012 and 19-2009R, APAFIS#7542-20 17030609233680). In the chronic colitis model, B6 mice received three oral cycles of 2% DSS (40 kDa; MP Biomedicals), followed by 7 days of regular drinking water. Ecto-TMEM219 was administered 0.1/mg/mouse daily i.p. from day 18 to day 32, then twice a week from day 35 to day 41 and at day 42 the animals were euthanized and/or subjected to endoscopy before sacrifice to confirm the inflammatory grade (9). All animal experiments were conducted in accordance with the Italian animal protection laws and were approved by the Italian Ministry of Health (No. 98/2022-PR and n. 1144/2020-PR).

Tmem219$^{flox/flox}$Lgr5^{cre} model

In order to demonstrate the effect of Tmem219 genetic ablation on intestinal stem cells, ISC-Tmem219$^{-/-}$ generated by breeding Tmem219$^{flox/flox}$ mice (10) with Lgr5^{cre} (B6.129P2-Lgr5tm1(cre/ERT2)Cle/J) mice were first injected with tamoxifen (20 mg/mL, T5648, Sigma Aldrich 100 µL, i.p., day -4 and -3) to induce Cre-mediated deletion of Tmem219 on LGR5-positive cells. ISC-Tmem219$^{-/-}$ mice in whom tamoxifen was not injected and the Cre/lox system was not activated were used as controls. For colitis induction, mice were administered DSS 2.5% in drinking water for 5 days, monitored for weight loss, DAI score, and sacrificed on day 12 for histological analysis and *in vitro* mechanistic studies. For the treatment study, tamoxifen was injected at the same dose on days 7 and 8 after the mice had completed the DSS treatment. We also induced enteritis in ISC-Tmem219$^{-/-}$ mice by injecting Polyinosinic:polycytidylic acid (poly I:C, # 31852-29-6, InVivoGen), a Toll-like receptor 3 activator, 20 ug/g per mouse i.p. at day 0 and harvesting after 36 hours (11).

Ex vivo analyses in murine models

Clinical and histological assessment of colitis in animal models

The Disease Activity Index (DAI) has been calculated based upon evaluation of body weight changes, stool consistency, and the presence of blood in the feces and in the anorectal area. Body weight (BW), stool consistency (with a score from 0 to 3: 0=normal, 1= soft, 2= Diarrhea, 3=watery Diarrhea), and visible presence of blood (rectum of mice) were recorded daily. At euthanasia, the presence of Occult Blood (OB) was recorded using the hemoccult method. Loss in BW was scored as: 0, no weight loss; 1, weight loss of <10% from baseline; 2, >10%. For stool consistency, a score of 0 was assigned for well-formed pellets, 1 for pasty and semi formed stools that did not adhere to the anus, and 2 for liquid stools that adhered to the anus. For OB, a score of 0 was assigned for no blood, 1 for positive OB or for gross bleeding. BW loss was calculated as the percentage difference between the original BW (day 0) and the BW on any day. To assess the level of inflammation, colon samples embedded in paraffin were stained with Hematoxylin and Eosin and analyzed. A multiparametric scoring (0 to 18) was performed by analyzing and grading the severity and extent of inflammation, the intensity of cellular infiltrate in the mucosa, its extension in sub-mucosa layers, and the presence of epithelial lesions. Finally, in the chronic colitis model mice were anesthetized at day 42 with a mixture of 80 mg/kg Ketamine and 5 mg/kg Xylazine and underwent endoscopy analysis to confirm the inflammatory grade by using the "Coloview system", including a miniature endoscope (scope 1.9 mm outer diameter), a xenon light source, a triple chip camera, and an air pump (Karl Storz, Tuttlingen, Germany). The endoscopic procedure was observed on a color monitor and a modified murine endoscopic index score of colitis severity was calculated based on colon translucency (0–3 points), granular features of the mucosa (0–3 points), morphology of the vascular pattern (0–4 points), and the presence of fibrin (0–4 points), with a

cumulative score ranging between 0 (no signs of inflammation) and 14 (endoscopic signs of very severe inflammation) as previously described (9).

Murine crypts isolation

Crypts were extracted from murine colon samples obtained from mice treated with different compounds. Briefly, colon samples were flushed with Hanks Balanced Salt Solution (HBSS) to remove mucus and cut longitudinally. Tissue was next minced into small pieces and incubated in 10 ml HBSS and vortexed. Samples were then transferred into 20 mM EDTA plus HBSS 15 ml tubes and incubated for 30 minutes at 37°C. A vigorous shaking of the sample next yielded supernatants enriched in colonic crypts. FBS (Sigma) was added to a final concentration of 10% and centrifuged 3 minutes at 800 rpm. Supernatant enriched in crypts was then removed carefully and used for flow cytometric analysis and flow sorting procedure, for qRT-PCR, for mini-guts assay.

Ex vivo murine mini-guts generation

Colon crypts obtained as described above were mixed with Matrigel and plated on pre-warmed culture dishes. After solidification, crypts were overlaid with complete culture medium consisting of Wnt3a-conditioned medium and Advanced DMEM/F12 (Life Technologies) 50:50, supplemented with L-glutamine 2mM, 10 mM HEPES, N-2 [1×], 10 mM Nicotinamide, 1 mM N-Acetyl-L-cysteine, 50 ng/ml murine EGF (Life Technologies), 1 μg/ml RSPO1 (Sino Biological), 100 ng/ml murine Noggin (Peprotech). Medium was replaced every 3 days. After 8 days of culture, mini-guts growth was measured and those with at least 1 visible crypt domain were considered as developed organoids.

Flow cytometry

Dissociated murine crypts were stained for flow cytometric analysis with the following antibodies: BB700 rat anti-mouse CD45 (clone 30-F11, 566439, BD Biosciences), Super Bright™ 600 rat anti-mouse EpCam (clone G8.8, 63-5791-82, Thermofisher), PE rat anti-mouse anti-CD24 (12-0242-82, Thermofisher), superbright 702 rat anti-mouse anti-CD44 (67-0441-82, Thermofisher), human anti-TMEM219 (courtesy provided by Yumab GmbH, Braunschweig, Germany). For TMEM219 staining, the human anti-TMEM219 (50 ug/ml) was used as primary antibody and then cells were incubated with the secondary polyclonal antibody PE goat anti-human IgG (12-499-82 Thermofisher).

Flow sorting

To confirm the genetic ablation of Tmem219 in the ISC-Tmem219$^{-/-}$ mouse, colonic crypts dissociated as above were processed to obtain single cell suspension for flow sorting. Cells were first stained with BB700 rat anti-mouse CD45 (clone 30-F11, 566439, BD Biosciences) to exclude leukocytes. Flow sorting was performed by using a Beckman Coulter MoFlo Astrios EQ and by gating on CD45$^-$ cells, and both CD45$^-$Lgr5EGFP$^+$and CD45$^-$Lgr5EGFP$^-$ cell fractions were collected for qRT-PCR analysis.

Serum and tissue analysis

IGF-I levels in treated and untreated mice were assessed using commercially available ELISA kit, according to the manufacturer's instructions (R&D Systems [Minneapolis, MN], #MG100). Caspase 8 expression were analyzed by ELISA (MyBiosource MBS2702600).

Histopathological and immunohistochemical methods

The various samples were fixed in a 10% buffered formalin solution and routinely embedded in paraffin tissue blocks from which 3-5-µm-thick histological sections were obtained from each case. One slide was stained with hematoxylin and eosin (H&E) for general morphological evaluation,

while additional unstained slides were used for immunohistochemical analysis. In short, sections were dewaxed in xylene, rehydrated through graded alcohols, and after inhibition of the endogenous peroxidase with a 3% H_2O_2 water solution, the specimens were incubated with a protein block (Ready to Use Dako Biotin Blocking System, Carpenteria, CA, USA). The human anti-TMEM219 primary antibody (courtesy provided by Yumab GmbH, Braunschweig, Germany) was tested in human intestinal samples, whereas the anti-IGFBP3 primary antibody (polyclonal, 1:50 dilution, Sigma Aldrich) was immunohistochemically tested in liver biopsies of patients with or without Crohn's disease (files stored at the Unit of Pathology of the Department of Biomedical, Biotechnological, and Translational Sciences, University of Parma, Parma, Italy). The reaction was revealed using the streptavidin-HRP Dako LSAB2 System, (K0675, Dako, Carpenteria, CA) and a 0.25% solution of 3,3′-Diaminobenzidine (DAB) tetrahydrochloride. Finally, the histological sections were counterstained with Harris hematoxylin. The following antibodies were also used to detect proliferation and stem cells: MKI67 (monoclonal, clone MIB1, 1:100 dilution, Dako, Carpinteria, CA, USA) and aldehyde dehydrogenase (monoclonal, clone 44/ALDH, 1:1000 dilution, 61194, BD Transduction Laboratories, Franklin Lakes, NJ, USA).

Immunofluorescence

Intestinal samples obtained from patients with/without Crohn's disease were evaluated by immunofluorescence with a confocal system (TCS SP2 Laser Scanning Confocal, Leica, Wetzlar, Germany). Briefly, after being fixed in buffered formalin (formaldehyde 4% w/v and acetate buffer 0.05 M) and processed in paraffin wax, 3um-thick sections of tissue were obtained and rehydrated. After being passed in xylene, ethanol 100%, 95 %, 70% and 50%, slides were rinsed with deionized water and washed with PBS 3 times and permeabilized with PBS containing 2% BSA and 0.3% Triton X-100 for 20 min. To block unspecific staining between the primary antibodies and the

tissue, slides were incubated with blocking buffer (10% Donkey serum in PBS) for 30 minutes at room temperature. Primary antibodies were diluted in PBS 1% donkey serum, incubated overnight, washed, and incubated with secondary antibodies. After washing, a 300 ul diluted solution of DAPI was added. Images were acquired in multitrack mode, using consecutive and independent optical pathways. The following primary antibodies were used for staining of human tissues: rabbit polyclonal TMEM219 (1:100 or 1:50, HPA059185, Sigma), mouse cytokerain 20 (1:100, monoclonal, clone Ks20.8, Dako), rabbit cytokeratin 20 (1:1, monoclonal, clone SP33, Ventana Medical Systems, Arizona, US) and human/mouse EphB2 antibody (1:50, MAB467 R&D Systems), mouse chromogranin (1:2, LK2H10 Ventana, Roche). CaCo2 cells and human purified crypts were incubated with IGFBP3 (50 ng/ml) and fixed in 10% neutral buffered for 30 min, washed with PBS 3 times and permeabilized with PBS containing 2% BSA and 0.3% Triton X-100 for 20 min, blocked with 10% serum, and then incubated with primary antibodies overnight at 4°C and subsequently labeled with fluorescent secondary antibodies for 2 h at room temperature. Primary and secondary antibodies were as following: rabbit polyclonal TMEM219 (1:100 or 1:50, HPA059185, Sigma), mouse monoclonal ALDH antibody (1:1000, clone 44, BD), mouse monoclonal IGFBP3 antibody (1:100, C45037, LSBio), donkey anti-mouse FITC (1:300) and donkey anti-rat TRITC (1:300) both from Jackson ImmunoResearch, West Grove, PA). Expression was analyzed using the ImageJ software, at least 3 pictures for each sample were evaluated and quantified using a semiquantitative score ranging from 1 to 5 (1=absent, 5=positive).

Generation of anti-IGFBP3 and anti-TMEM219 monoclonal antibody

The monoclonal anti-IGFBP3 antibody was generated by phage-display technology using a recombinant full length human IGFBP3 (R&D, 8874-B3) as antigen for the screening in collaboration with Yumab as already reported (10). The monoclonal anti-TMEM219 antibody was

generated by hybridoma technology using ecto-TMEM219 (Genescript), the extracellular portion of TMEM219 as antigen. Both mAbs were used *in vivo* 0.5 mg/mouse daily from day -3 to day 12 in a DSS prevention protocol.

SUPPLEMENTARY TABLES

Supplementary Table 1. Baseline demographic characteristics of human subjects enrolled in the clinical study (Figure 1 and 2).

	**CTRL (n =39)**	**Crohn's disease (n =112)**	***p value***
Age – yr	48.9 ± 2.8	46.6 ± 1.4	0.72
Male – n (%)	19 (48.7)	80 (71.4)	0.02
Disease duration – yr	-	14.0 ± 1.1	-
Disease status – n (%)	-		-
- *Active (clinical/endoscopic)*	-	39 (35)	-
- *Remission (endoscopic)*	-	34 (30)	-
- *No response to medical therapy (after 6 months)*		39 (35)	
Extraintestinal disease - n (%)	-	22 (20)	-
- *Musculoskeletal manifestations*	-	12 (0.1)	-
- *Mucocutaneous manifestations*	-	9 (0.1)	-
IBD treatment – n (%)			
Other (Mesalazin, steroids)	-	12 (11)	-
Anti-TNF agents	-	61 (54)	-
Immunosuppressant	-	12 (11)	-
Biological drugs (vedolizumab, ustekinumab)	-	27 (24)	-

Abbreviations. CTRL, control subjects without a diagnosis of Crohn's disease; yr, years. Data are expressed as mean ± standard error of mean (SEM). P value CTRL vs. Crohn's disease by Student t test with Welch correction or with Fisher test.

Supplementary Table 2. List of differentially expressed stem cell-related genes (fold change) identified by transcriptome profiling in freshly isolated samples obtained from controls (CTRL) as compared to patients with active Crohn's disease (marginal area and inflamed area), responder and non-responder patients (related to Figure 1).

Samples	Downregulated genes	Upregulated genes
**Patients with active disease (marginal area) vs. CTRL**	*HDAC2, NOTCH2, CDCA7, SLC12A2, HSPA9, DKC1, CCND2, ETS2, FAM84A, LGR5, GPX2*	*FOXA2, SOX2, WNT1*
**Patients with active disease (inflamed area) vs. CTRL**	*AXIN2, AXIN1, FOXA2, NOTCH1, BMI1, RNF43, BMP1, HDAC2, NOTCH2, CDCA7, SLC12A2, CCNA2, HSPA9, NUMB, CDK6, SOX9, CCND1, JAG1, SOX1, DKC1, ZNRF3, CCND2, KAT2A, SOX2, EPHB2, CCNE1, KAT8, TERT, LGR5, FGF1, MYC, WNT1, ALDH1A1, FGF2, NEUROG2*	
**Responder patients vs. CTRL**	*RNF43,NOTCH2, SLC12A2, SOX1, DKC1,ZNRF3, LGR5, GFG1, WNT1, ALDH1A, FGF2*	*OLFM4, BMI1*
**Non responder patients vs. CTRL**	*AXIN2, BMI1, RNF43, NOTCH2, SLC12A2, CCNA2, CDK6, JAG1, SOX1, DKC1, ZNRF3, SOX2, EPHB2, CCNE1, KAT8, TERT, LGR5, MYC, WNT1, ALDH1A1, FGF2*	*GPX2*
**Responder vs. non responder patients**		*AXIN2, BMI1, CCNA2, CDK6, SOX1, DKC1, SOX2, EPHB2, CCNE1, KAT8, LGR5, FGF1*

Supplementary Table 3. List of differentially expressed apoptosis-related genes (fold change) identified by transcriptome profiling in samples obtained from controls (CTRL) as compared to patients with active Crohn's disease (marginal area and inflamed area), responder and non-responder patients (related to Figure 2).

Samples	Downregulated genes	Upregulated genes
**Patients with active disease (marginal area) vs. CTRL**	*BIRC3, BIRC5, CASP4, CASP7, CASP9, DAPK1, FADD, FAS, HRK , MCL1, NFKB1, TNFRSF10A, TNFRSF11B, TNFRSF1B,TNFRSF21, TNFRSF9, TNFSF8*	*ABL1, BAG1, BAX, BCL10, BCL2L11, BID, BIRC6, BNIP2, BRAF, CASP2, CASP3, CASP5, CASP6, CASP8, CD27, CFLAR, CIDEA, CIDEB, DFFA, IGF1R, PYCARD, TNF, TNFRSF1A, TP53BP2, TRADD, XIAP*
**Patients with active disease (inflamed area) vs. CTRL**	*ABL1, BCL2, BCL2L10, BIRC3, CASP4, CD40LG, CYCS, FADD, TNFRSF9, TP53BP2*	*AKT1, BAK1, BAX, BCL10, BCL2A1, BCL2L11, BCL2L2, BFAR, BIK, BIRC5, BIRC6, BRAF, CASP10, CASP2, CASP3, CASP6, CASP7, CASP8, CASP9, CD27, CD70, CFLAR, GADD45A, PYCARD, RIPK2, TNFRSF1A, TNFSF10, TNFSF8, TP53*
**Responder patients vs. CTRL**	*BIRC3, BIRC5, BIRC6, CASP4, CASP7, CASP9, CIDEB, CRADD, CYCS, DAPK1, GADD45A, HRK, IL10, NFKB1, TNFRSF11B, TNFRSF21, TNFSF8, XIAP*	*AKT1, BAX, BCL10, BCL2, BCL2A1, BCL2L11, BFAR, BNIP2, BRAF, CASP1, CASP10, CASP14, CASP2, CASP3, CASP5, CASP8, CD27, CD70, CFLAR, DFFA, FASLG, IGF1R, LTBR, NOL3, PYCARD, RIPK2, TNFRSF1A, TNFRSF1B, TNFRSF25, TNFRSF9, TNFSF10, TP53, TP73, TRADD*
**Non responder patients vs. CTRL**	*BCL2, BIRC3, CASP9, CYCS, FADD, NFKB1, TNFRSF9, TNFSF8*	*ABL1, AKT1, BAD, BAG1, BAK1, BAX, BCL10, BCL2L10, BCL2L11, BFAR, BID, BIK, BIRC2, BIRC6, BNIP2, BRAF, CASP10, CASP14, CASP2, CASP3, CASP4, CASP6, CASP7, CASP8, CD27, CFLAR, CIDEA, CIDEB, DFFA, DIABLO, GADD45A, IGF1R, LTBR, PYCARD, TNF, TNFRSF1A, TNFRSF21, TNFSF10, TP53, TRADD, TRAF2, XIAP*
**Responder vs. non responder patients**	*ABL1, BAD, BAG1, BAK1, BAX, BCL2L10, BID, BIRC2, BIRC3, BIRC5, BIRC6, CASP4, CASP6, CASP7, CASP8, CASP9, CIDEA, CIDEB, CRADD, DAPK1, DIABLO, GADD45A, HRK, IGF1R, IL10, PYCARD, TNF, TNFRSF21, TNFSF8, TRADD, TRAF2, XIAP*	*BCL10, BCL2, BCL2A1, BNIP2, BRAF, CASP10, CASP3, CASP5, CD40LG, CD70, FADD, FASLG, NOL3, RIPK2, TNFRSF1B, TNFRSF9, TP73*

Supplementary Table 4. List of top 100 genes interacting with Caspase 8 identified by using Genemania Cytoscape bioinformatic approach (related to Figure 2).

Gene Description	Rank
CASP8 caspase 8 [Source:HGNC Symbol;Acc:HGNC:1509] N/A	
CFLAR CASP8 and FADD like apoptosis regulator [Source:HGNC Symbol; Acc:HGNC:1876]	1
FAS Fas cell surface death receptor [Source:HGNC Symbol;Acc:HGNC: 11920]	2
DNM1L dynamin 1 like [Source:HGNC Symbol;Acc:HGNC:2973]	3
FADD Fas associated via death domain [Source:HGNC Symbol;Acc:HGNC: 3573]	4
BAX BCL2 associated X, apoptosis regulator [Source:HGNC Symbol;Acc: HGNC:959]	5
TNFRSF10B TNF receptor superfamily member 10b [Source:HGNC Symbol;Acc: HGNC:11905]	6
NMT1 N-myristoyltransferase 1 [Source:HGNC Symbol;Acc:HGNC:7857]	7
RFFL ring finger and FYVE like domain containing E3 ubiquitin protein ligase [Source:HGNC Symbol;Acc:HGNC:24821]	8
TNFSF10 TNF superfamily member 10 [Source:HGNC Symbol;Acc:HGNC: 11925]	9
FASLG Fas ligand [Source:HGNC Symbol;Acc:HGNC:11936]	10
CASP10 caspase 10 [Source:HGNC Symbol;Acc:HGNC:1500]	11
TRADD TNFRSF1A associated via death domain [Source:HGNC Symbol;Acc: HGNC:12030]	12
RNF34 ring finger protein 34 [Source:HGNC Symbol;Acc:HGNC:17297] 13	13
BIRC3 baculoviral IAP repeat containing 3 [Source:HGNC Symbol;Acc: HGNC:591]	14
BCAP31 B cell receptor associated protein 31 [Source:HGNC Symbol;Acc: HGNC:16695]	15
BID BH3 interacting domain death agonist [Source:HGNC Symbol;Acc: HGNC:1050]	16
TNFRSF10A TNF receptor superfamily member 10a [Source:HGNC Symbol;Acc: HGNC:11904]	17
PLEC plectin [Source:HGNC Symbol;Acc:HGNC:9069]	18
BIRC2 baculoviral IAP repeat containing 2 [Source:HGNC Symbol;Acc: HGNC:590]	19
FAM185A family with sequence similarity 185 member A [Source:HGNC 20 Symbol;Acc:HGNC:22412]	20
RIPK1 receptor interacting serine/threonine kinase 1 [Source:HGNC Symbol; Acc:HGNC:10019]	21
NOD1 nucleotide binding oligomerization domain containing 1 [Source: HGNC Symbol;Acc:HGNC:16390]	22
IFIH1 interferon induced with helicase C domain 1 [Source:HGNC Symbol; Acc:HGNC:18873]	23
MLKL mixed lineage kinase domain like pseudokinase [Source:HGNC Symbol;Acc:HGNC:26617]	24
SPP1 secreted phosphoprotein 1 [Source:HGNC Symbol;Acc:HGNC:11255] 25	25
TRAF2 TNF receptor associated factor 2 [Source:HGNC Symbol;Acc:HGNC: 12032]	26
PEA15 proliferation and apoptosis adaptor protein 15 [Source:HGNC Symbol;Acc:HGNC:8822]	27
MAP3K14 mitogen-activated protein kinase kinase kinase 14 [Source:HGNC Symbol;Acc:HGNC:6853]	28
ATG4D autophagy related 4D cysteine peptidase [Source:HGNC Symbol;Acc: HGNC:20789]	29
TNFAIP3 TNF alpha induced protein 3 [Source:HGNC Symbol;Acc:HGNC: 11896]	30

UBE2D2 ubiquitin conjugating enzyme E2 D2 [Source:HGNC Symbol;Acc: HGNC:12475]	31
TICAM1 toll like receptor adaptor molecule 1 [Source:HGNC Symbol;Acc: HGNC:18348]	32
SERPINB9 serpin family B member 9 [Source:HGNC Symbol;Acc:HGNC:8955] 33	33
MAVS mitochondrial antiviral signaling protein [Source:HGNC Symbol;Acc: HGNC:29233]	34
NOL3 nucleolar protein 3 [Source:HGNC Symbol;Acc:HGNC:7869]	35
RNF135 ring finger protein 135 [Source:HGNC Symbol;Acc:HGNC:21158]	36
TRIM25 tripartite motif containing 25 [Source:HGNC Symbol;Acc:HGNC: 12932]	37
TICAM2 toll like receptor adaptor molecule 2 [Source:HGNC Symbol;Acc: HGNC:21354]	38
UBE2D3 ubiquitin conjugating enzyme E2 D3 [Source:HGNC Symbol;Acc: HGNC:12476]	39
VANGL1 VANGL planar cell polarity protein 1 [Source:HGNC Symbol;Acc: HGNC:15512]	40
TLR3 toll like receptor 3 [Source:HGNC Symbol;Acc:HGNC:11849]	41
GMNN geminin DNA replication inhibitor [Source:HGNC Symbol;Acc: HGNC:17493]	42
DDX58 DExD/H-box helicase 58 [Source:HGNC Symbol;Acc:HGNC:19102] 43	43
DEDD2 death effector domain containing 2 [Source:HGNC Symbol;Acc: HGNC:24450]	44
PCYT1A phosphate cytidylyltransferase 1, choline, alpha [Source:HGNC Symbol;Acc:HGNC:8754]	45
IKBKB inhibitor of nuclear factor kappa B kinase subunit beta [Source: HGNC Symbol;Acc:HGNC:5960]	46
VIM vimentin [Source:HGNC Symbol;Acc:HGNC:12692]	47
CHUK component of inhibitor of nuclear factor kappa B kinase complex [Source:HGNC Symbol;Acc:HGNC:1974]	48
TLR4 toll like receptor 4 [Source:HGNC Symbol;Acc:HGNC:11850]	49
BLM BLM RecQ like helicase [Source:HGNC Symbol;Acc:HGNC:1058]	50
LY96 lymphocyte antigen 96 [Source:HGNC Symbol;Acc:HGNC:17156]	51
PIAS1 protein inhibitor of activated STAT 1 [Source:HGNC Symbol;Acc: HGNC:2752]	52
IKBKG inhibitor of nuclear factor kappa B kinase regulatory subunit gamma [Source:HGNC Symbol;Acc:HGNC:5961]	53
CD14 CD14 molecule [Source:HGNC Symbol;Acc:HGNC:1628] 54	54
BFAR bifunctional apoptosis regulator [Source:HGNC Symbol;Acc:HGNC: 17613]	55
DEDD death effector domain containing [Source:HGNC Symbol;Acc:HGNC: 2755]	56
CYP2W1 cytochrome P450 family 2 subfamily W member 1 [Source:HGNC Symbol;Acc:HGNC:20243]	57
LY9 lymphocyte antigen 9 [Source:HGNC Symbol;Acc:HGNC:6730]	58
HSH2D hematopoietic SH2 domain containing [Source:HGNC Symbol;Acc: HGNC:24920]	59
CASP8AP2 caspase 8 associated protein 2 [Source:HGNC Symbol;Acc:HGNC: 1510]	60
JMJD7-PLA2G4B readthrough [Source:HGNC Symbol;Acc:HGNC: 34449]	61
PYCARD PYD and CARD domain containing [Source:HGNC Symbol;Acc: HGNC:16608]	62
ZAP70 zeta chain of T cell receptor associated protein kinase 70 [Source: HGNC Symbol;Acc:HGNC:12858]	63
CASP14 caspase 14 [Source:HGNC Symbol;Acc:HGNC:1502]	64
AR androgen receptor [Source:HGNC Symbol;Acc:HGNC:644]	65
DHX34 DExH-box helicase 34 [Source:HGNC Symbol;Acc:HGNC:16719]	66
NUCB2 nucleobindin 2 [Source:HGNC Symbol;Acc:HGNC:8044]	67
CD3E CD3e molecule [Source:HGNC Symbol;Acc:HGNC:1674]	68
TMEM219 transmembrane protein 219 [Source:HGNC Symbol;Acc:HGNC: 25201]	69
UBE2D1 ubiquitin conjugating enzyme E2 D1 [Source:HGNC Symbol;Acc: HGNC:12474]	70

EDA2R ectodysplasin A2 receptor [Source:HGNC Symbol;Acc:HGNC:17756]	71
ILK integrin linked kinase [Source:HGNC Symbol;Acc:HGNC:6040]	72
SERTAD2 SERTA domain containing 2 [Source:HGNC Symbol;Acc:HGNC: 30784]	73
PACSIN2 protein kinase C and casein kinase substrate in neurons 2 [Source: HGNC Symbol;Acc:HGNC:8571]	74
ZNF707 zinc finger protein 707 [Source:HGNC Symbol;Acc:HGNC:27815] 75	75
DENND2D DENN domain containing 2D [Source:HGNC Symbol;Acc:HGNC: 26192]	76
CXCL5 C-X-C motif chemokine ligand 5 [Source:HGNC Symbol;Acc:HGNC: 10642]	77
SAMSN1 SAM domain, SH3 domain and nuclear localization signals 1 [Source: HGNC Symbol;Acc:HGNC:10528]	78
ITGA4 integrin subunit alpha 4 [Source:HGNC Symbol;Acc:HGNC:6140]	79
MYO1G myosin IG [Source:HGNC Symbol;Acc:HGNC:13880]	80
ORC2 origin recognition complex subunit 2 [Source:HGNC Symbol;Acc: HGNC:8488]	81
HECTD3 HECT domain E3 ubiquitin protein ligase 3 [Source:HGNC Symbol; Acc:HGNC:26117]	82
MX2 MX dynamin like GTPase 2 [Source:HGNC Symbol;Acc:HGNC:7533] 83	83
PRDM8 PR/SET domain 8 [Source:HGNC Symbol;Acc:HGNC:13993] 84	84
CASP7 caspase 7 [Source:HGNC Symbol;Acc:HGNC:1508] 85	85
GMEB1 glucocorticoid modulatory element binding protein 1 [Source:HGNC Symbol;Acc:HGNC:4370]	86
TLR9 toll like receptor 9 [Source:HGNC Symbol;Acc:HGNC:15633] 87	87
MAP4K1 mitogen-activated protein kinase kinase kinase kinase 1 [Source: HGNC Symbol;Acc:HGNC:6863]	88
PROZ protein Z, vitamin K dependent plasma glycoprotein [Source:HGNC Symbol;Acc:HGNC:9460]	89
STT3A STT3 oligosaccharyltransferase complex catalytic subunit A [Source: HGNC Symbol;Acc:HGNC:6172]	90
NR1H4 nuclear receptor subfamily 1 group H member 4 [Source:HGNC Symbol;Acc:HGNC:7967]	91
UPK3B uroplakin 3B [Source:HGNC Symbol;Acc:HGNC:21444] 92	92
STAT4 signal transducer and activator of transcription 4 [Source:HGNC Symbol;Acc:HGNC:11365]	93
MIER1 MIER1 transcriptional regulator [Source:HGNC Symbol;Acc:HGNC: 29657]	94
SH3BGRL SH3 domain binding glutamate rich protein like [Source:HGNC Symbol;Acc:HGNC:10823]	95
TIAF1 TGFB1-induced anti-apoptotic factor 1 [Source:HGNC Symbol;Acc: HGNC:11803]	96
FZD6 frizzled class receptor 6 [Source:HGNC Symbol;Acc:HGNC:4044]	97
ARHGAP45 Rho GTPase activating protein 45 [Source:HGNC Symbol;Acc: HGNC:17102]	98
SUMO1 small ubiquitin like modifier 1 [Source:HGNC Symbol;Acc:HGNC: 12502]	99
CRHBP corticotropin releasing hormone binding protein [Source:HGNC Symbol;Acc:HGNC:2356]	100

1

Supplementary Table 5. List of all phosphorylated proteins and antibodies used for detection in the phospho-proteomic analysis (related to Figure 3).

Supplementary Table 6. Main characteristics of primers used in qRT-PCR analysis.

Gene Symbol	Refseq Accession #	Band Size (bp)	Reference Position
Human			
TMEM219	NM_ 001083613.1	60	726
LGR5	NM__003667	91	1665
EPHB2	NM_004442	68	2908
CASP8	NM_ 001080124.1	124	648
ACTB	NM_001101	174	730
Murine			
Tmem219	NM_026827.1	78	677
Lgr5	NM_010195.2	64	571
Ephb2	NM_010142.2	85	1696
Casp8	NM_001080126.1	96	1525
Mki67	NM_001081117.2	73	1776
Aldh1a1	NM_013467.3	538	116
Il-22ra1	NM_178257.2	69	591
Hprt	NM_013556.2	81	276

Abbreviations: Refseq, reference sequence.

SUPPLEMENTARY FIGURES

Supplementary Figure 1. **Intestinal stem cells defect in active Crohn's disease is linked to a TMEM219-Caspase 8 interplay**.

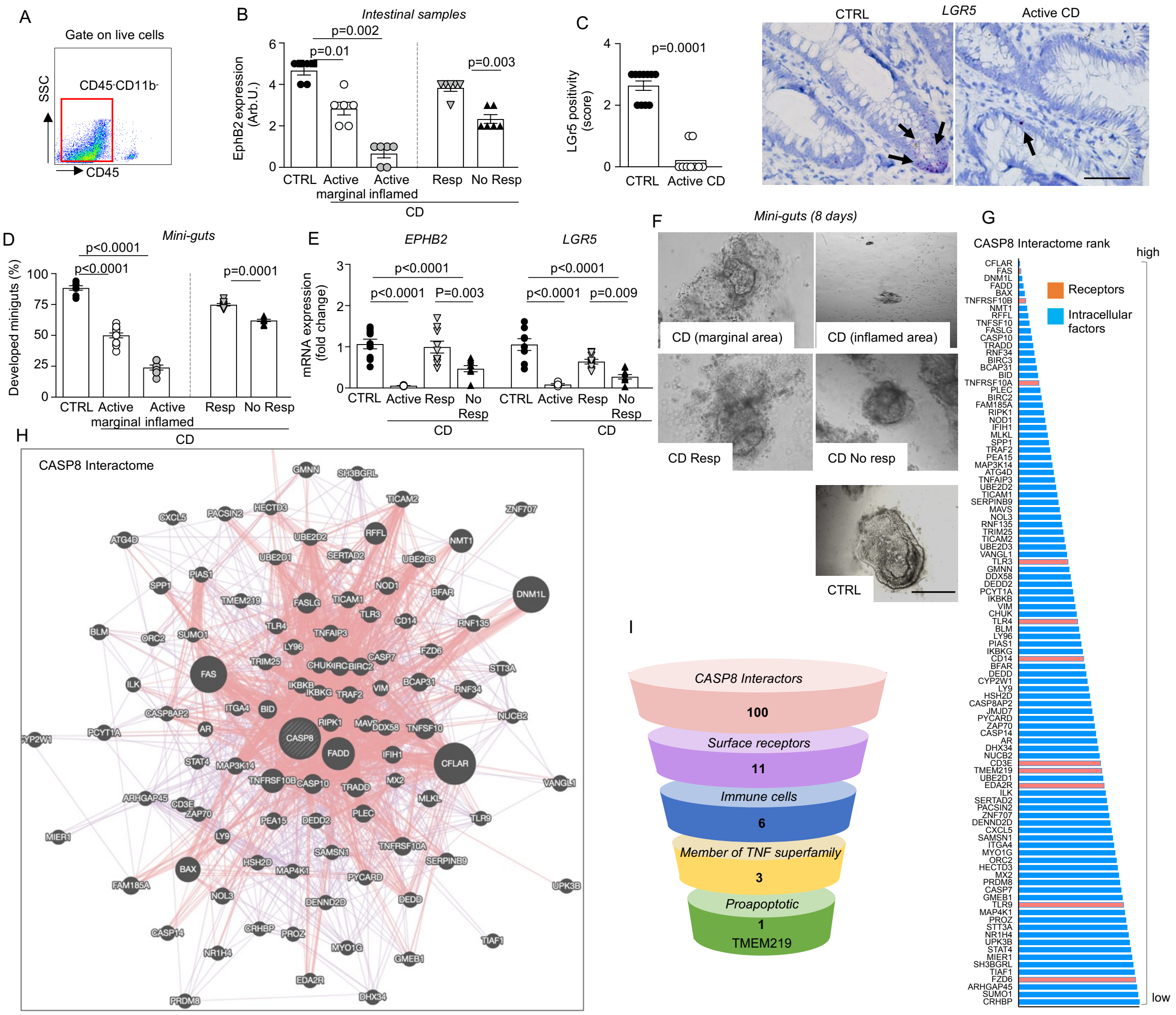

(A). Flow cytometry gating strategy for analysis of EPHB2 expression in Figure 1A. **(B)**. Quantification of EPHB2 expression at the confocal analysis in intestinal samples of controls (CTRL), patients with active Crohn's disease (CD, marginal and inflamed areas), responder in remission phase (Resp) and non-responder patients (No Resp), (n=3 pictures/sample were analyzed). **(C)**. Quantification and representative pictures of LGR5 expression detected by in situ hybridization in samples of patients with active CD disease as compared to healthy controls (n=3/group). Original magnification 20×, scale bar 50 µm. **(D)**. Development of 8-day crypts organoids (mini-guts) obtained from controls and from patients with Crohn's disease of all patient cohorts (n=8-12). Original magnification 20×, scale bar 100 µm. **(E)**. Normalized mRNA

expression of the ISC markers *EPHB2* and *LGR5* by qRT-PCR quantified in samples as in C. **(F)**. Representative pictures of organoids analyzed in C. **(G**, **H)**. Rank and network of Caspase 8 gene-gene interactions generated by the Genemania tool, based on molecular function and physical associations. Up to 100 most related genes are shown. Blue: intracellular factors. Orange: membrane receptors. **(I)**. Schematic representation showing the selection process of TMEM219 as signal to be explored in a Caspase 8-mediated ISCs damage in Crohn's disease. Mean ± standard error of the mean unless otherwise reported. At least three independent experiments performed in duplicate. One-way ANOVA followed by Sidak post-hoc analysis or unpaired Student t-test or Mann-Whitney t-test.

Supplementary Figure 2. **Caspase-8-mediated TMEM219 signaling in Crohn's disease**.

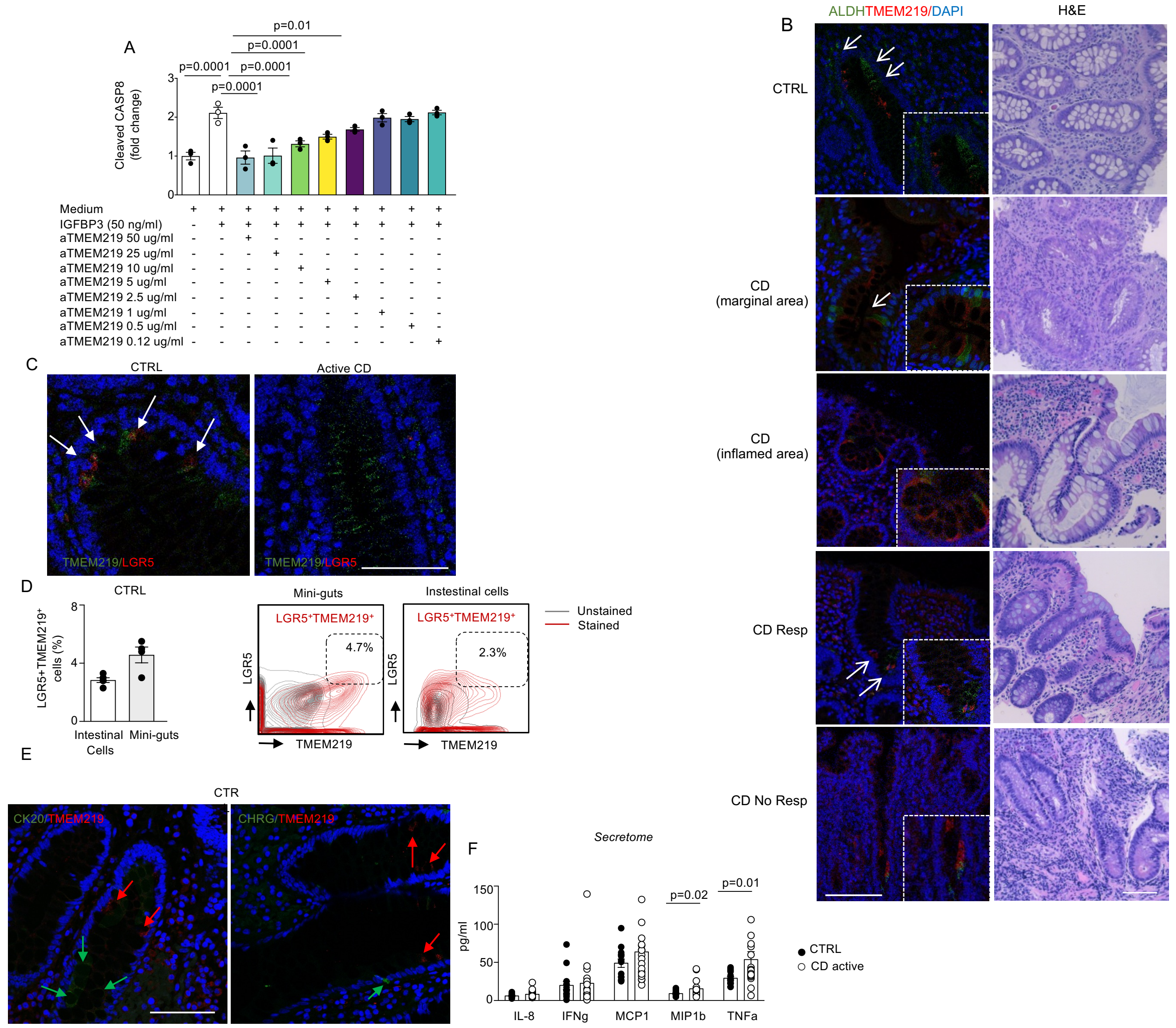

(A). Bar graphs quantifying Cleaved Caspase 8 (fold change) in CaCo2 cell line cultured with/without IGFBP3 (50 ng/ml) and in the presence/absence of TMEM219 inhibitor in a dose response assay. **(B)**. Representative pictures of the ISC marker ALDH (green) and TMEM219 (red) co-expression at the confocal analysis in intestinal samples of controls (CTRL), of patients with active disease from marginal and inflamed areas, responder patients in the remission phase (Resp) and non-responder (No Resp) patients. Nuclei stained by DAPI. H&E staining of the corresponding sample is reported on the right side of each picture. Original magnification 20×, scale bar 100 µm. **(C**, **D)**. Representative picture and bar graph of LGR5 and TMEM219 co-expression performed in intestinal samples and in organoids of controls and analyzed by confocal analysis and flow cytometry (n=4-5). **(E)**. Representative picture of confocal analysis showing co-expression of TMEM219 (red) with enterocyte marker CK20 (green) and enteroendocrine marker CHRG (green) in intestinal samples of controls. In C and E: original magnification 20×, scale bar 50 µm. **(F)**.

Secretome profile analyzed by Luminex in serum collected from controls (n=14) and from patients with active Crohn's disease (n=19). At least three independent experiments performed in duplicates. Data are expressed as mean ± standard error of the mean unless otherwise reported. One-Way ANOVA followed by Sidak post hoc test, two-sided t test.

Supplementary Figure 3. **Mechanistic studies on IGFBP3/TMEM219 axis**.

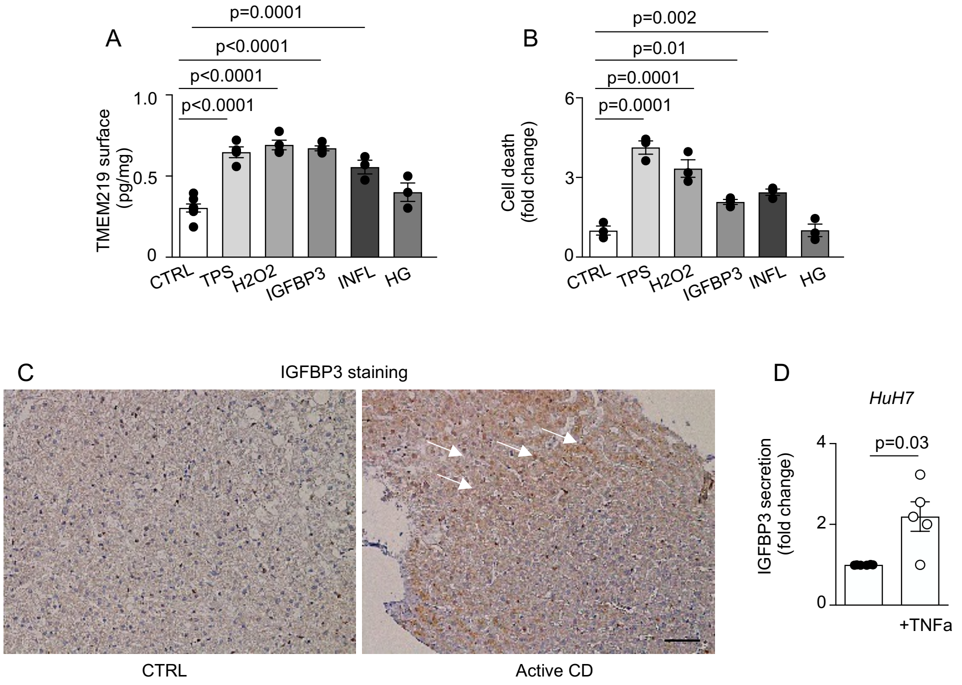

(A, **B)**. Quantification of TMEM219 expression and cell death by ELISA in CACO2 cells cultured in the following conditions: IGFBP3 (50 ng/ml), Glucose 35 mM, H_2O_2 400 mM, Thapsigargin 3 µm and a cocktail of cytokines (IL-1b, 20 ng/ml, IFNg, 10 ng/ml, IL-6, 20 ng/ml and TNFa 10 ng/ml), (n=3 independent experiments run in triplicates). **(C)**. Representative picture of IGFBP3 detection by immunohistochemistry in human liver samples obtained from patients with/without Crohn's disease (n=3 samples were analyzed). 20×, scale bar 100 µm. **(D)**. Quantification of IGFBP3 in the supernatant of HuH7 cultured in the presence/absence of TNF-alpha for 72 hours. Data are expressed as mean ± standard error of the mean (SEM) unless otherwise reported. Statistical analysis was performed by two-sided Student t test and one-Way ANOVA followed by Sidak post hoc test.

Supplementary Figure 4. **Mechanistic studies on TMEM219 signaling pathway**.

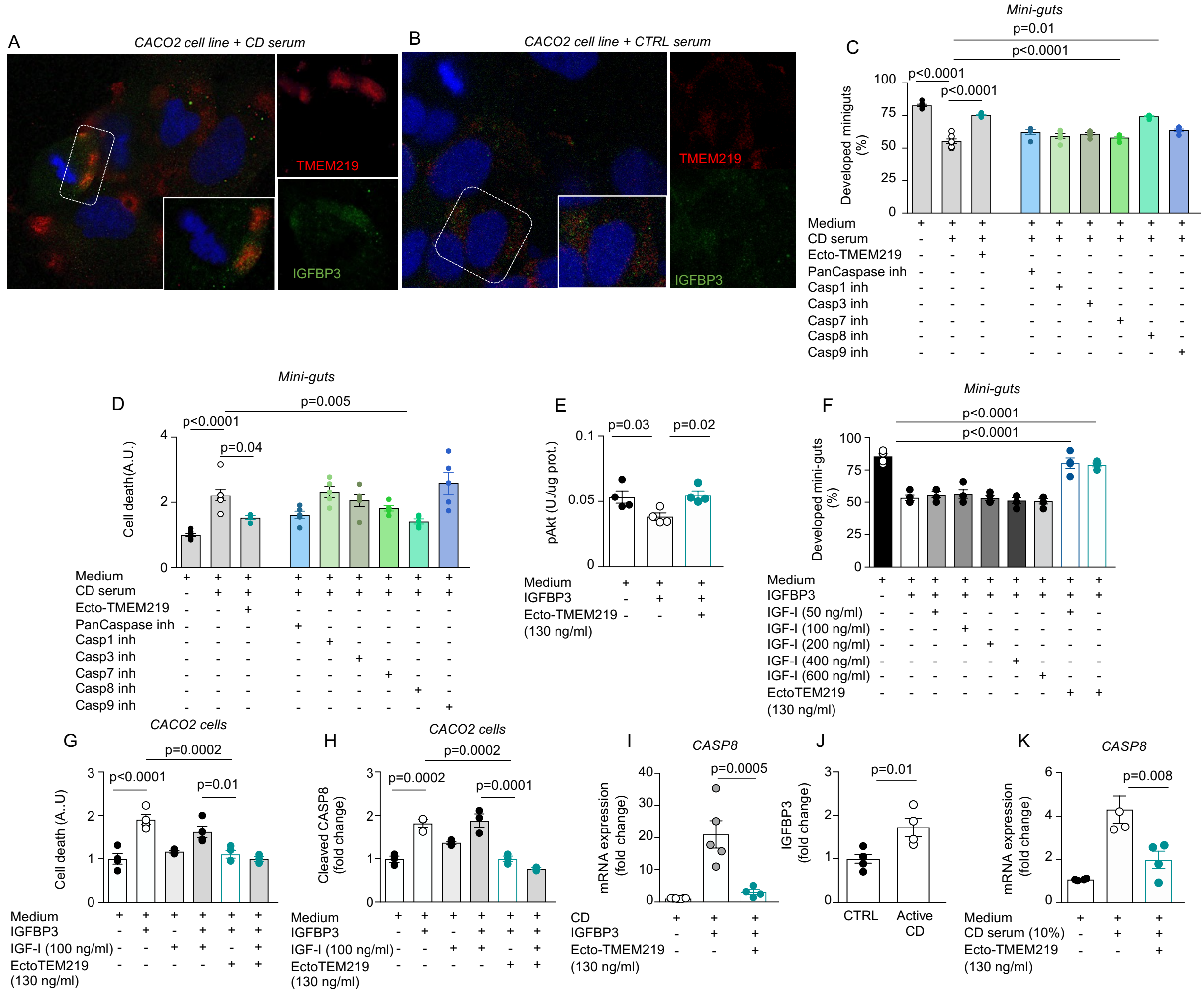

(A, **B)**. Confocal microscopy analysis (scale bar 10 µm, 63× original magnification) depicting colocalization and binding of TMEM219 (red) and IGFBP3 (green) in CaCo2 cells cultured with pooled sera of patients with active Crohn's disease or controls. Cells were stained with DAPI for nuclei (blue) and immunolabeled with anti-TMEM219 (red) and anti-IGFP3 Abs (green). **(C**, **D)**. Cell death and development quantified in mini-guts of controls and cultured with pooled serum of patients with active Crohn's disease in place of 10% FBS and with ecto-TMEM219, Pan Caspase inhibitor and Caspases selective inhibitors (Caspases 1, 3, 7, 8 and 9) (n=6/8). **(E)**. Phosphorylated-Akt (pAkt) quantified in CaCo2 cells cultured with/without IGFBP3 and with/without ecto-TMEM219 (n=4). **(F)**. Development of mini-guts of controls cultured +/- IGFBP3, IGF-I at increasing concentrations and +/- Ecto-TMEM219 (n=4). **(G**, **H)**. Cell death and Cleaved Caspase 8 measured in CACO2 cells cultured +/- IGFBP3, IGF-I and +/- Ecto-TMEM219. **(I**, **K)**. *CASP8* normalized mRNA expression in mini-guts of patients with active disease cultured with/without IGFBP3 and ecto-TMEM219 and in mini-guts obtained from crypts of controls and cultured with pooled Crohn's disease serum, with/without ecto-TMEM219 (n=4-7). **(J)**. IGFBP3 expression in intestinal samples of patients with active Crohn's disease as compared to controls (n=4). Mean ±

standard error of the mean. At least three independent experiments run in duplicate. One-way ANOVA followed by Sidak's post-hoc test and two-sided t-test.

Supplementary Figure 5. Pharmacological blockade of IGFBP3/TMEM219 signal ameliorates DSS-mediated acute colitis *in vivo* in a preventive and treatment model.

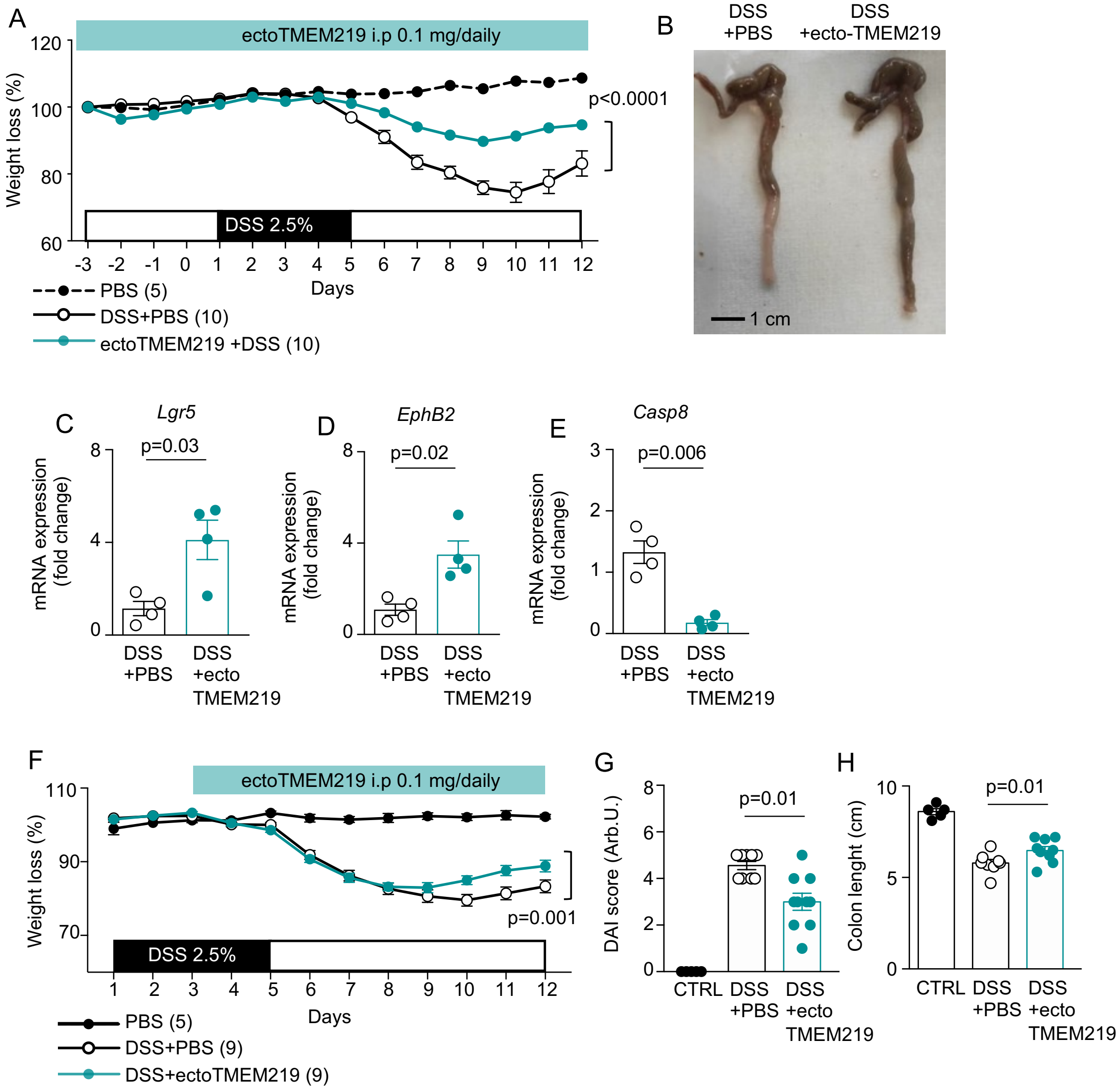

(A). Weight loss percentage measured in B6 mice receiving or not oral DSS (dextran sulfate sodium 2.5%, 5 days), and treated with ecto-TMEM219 (0.1 mg/day/mouse daily starting at day -3) or vehicle (PBS) in a prevention study (n=10, controls n=5). **(B)**. Representative pictures of colons harvested at day 12 in DSS+PBS and DSS+ecto-TMEM219 treated mice. Scale bar 1 cm. **(C, D, E)**. Normalized mRNA expression of *Ephb2, Lgr5* and *Casp8* in colon of DSS+PBS as compared to DSS+ecto-TMEM219 treated mice (n=4). **(F)**. Weight loss percentage measured in B6 mice receiving or not receiving oral DSS and treated with ecto-TMEM219 (0.1 mg/day/mouse daily from day +3) or PBS in a curative setting (n=10, controls n=5). **(G, H)**. DAI score and colon length measured at day 12 in controls (n=5), in DSS+PBS and DSS+ecto-TMEM219 treated mice (n=10). Mean ± standard error of the mean. Two-way or One-way ANOVA followed by Sidak's post-hoc test and two-sided t-test.

Supplementary Fig. 6. Direct inhibition of TMEM219 and IGFBP3 in DSS acute colitis model.

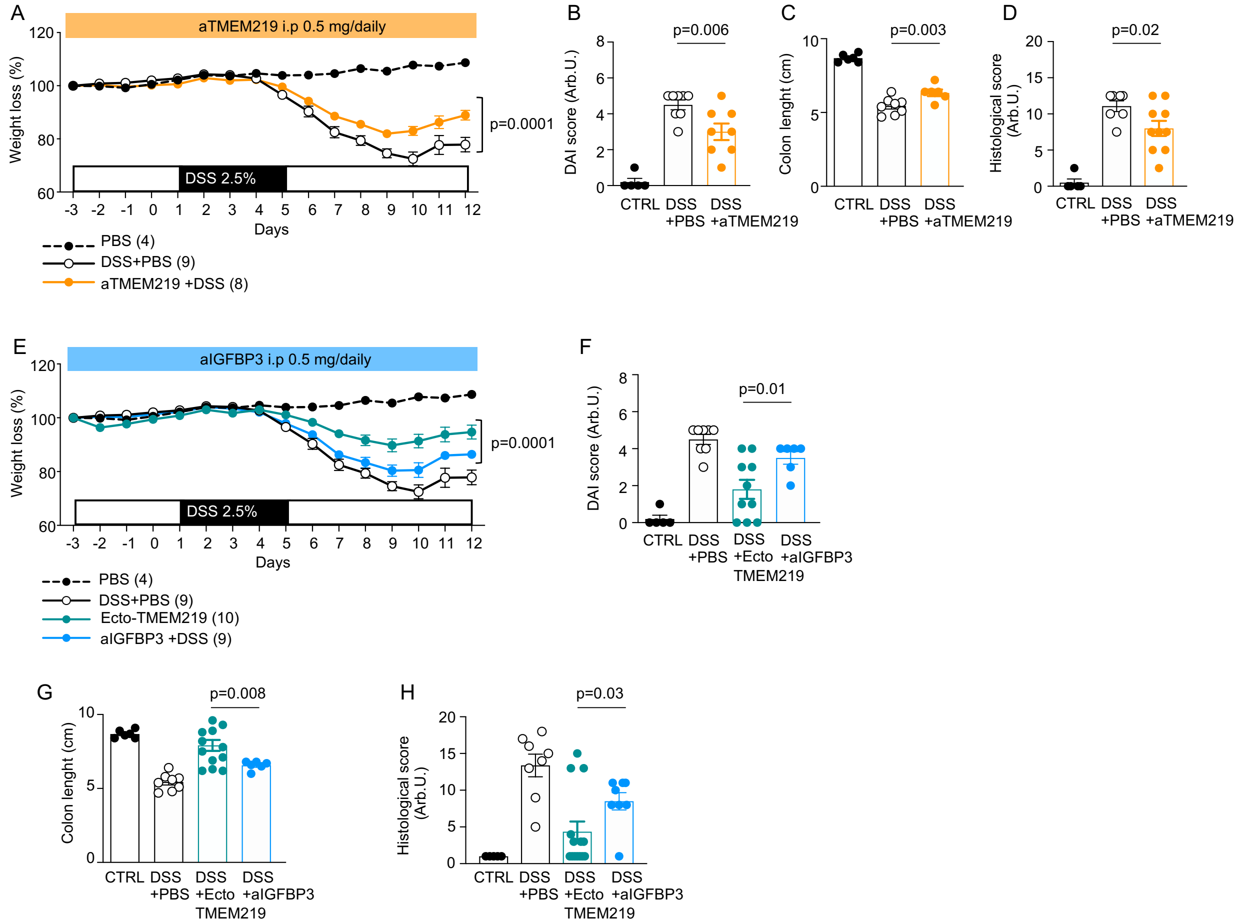

(A). Weight loss percentage measured in B6 mice receiving or not oral DSS (dextran sulfate sodium 2.5%, 5 days), and treated with anti-TMEM219 monoclonal antibody (0.5 mg/day/mouse daily starting at day -3, n=8) or vehicle (PBS, n=9) in a prevention study (controls n=5). **(B, C, D)**. DAI score (n=8/group), colon length (n=6-7) and histological score (n=8/group) measured at day 12 in controls (n=5), in DSS+PBS and DSS+anti-TMEM219 treated mice. **(E)**. Weight loss percentage measured in B6 mice receiving or not oral DSS (dextran sulfate sodium 2.5%, 5 days), and treated with anti-IGFBP3 monoclonal antibody (0.5 mg/day/mouse daily starting at day -3) or vehicle (PBS) in a prevention study (n=9/group, controls n=4). **(F, G, H)**. DAI score (n=6-8), colon length (n=6-8/group) and histological score (n=8/group) measured at day 12 in controls (n=4-6), in DSS+PBS and DSS+anti-IGFBP3 treated mice. A group of DSS+Ecto-TMEM219-treated mice has been included in panels E, F, G and H for comparison (n=10 DAI, n=13 colon length, n=14 histological score). Mean ± standard error of the mean. Two-way or One-way ANOVA followed by Sidak's post-hoc test and two-sided Student t-test.

Supplementary Figure 7. Pharmacological blockade of IGFBP3/TMEM219 signal ameliorates DSS-mediated chronic colitis *in vivo*.

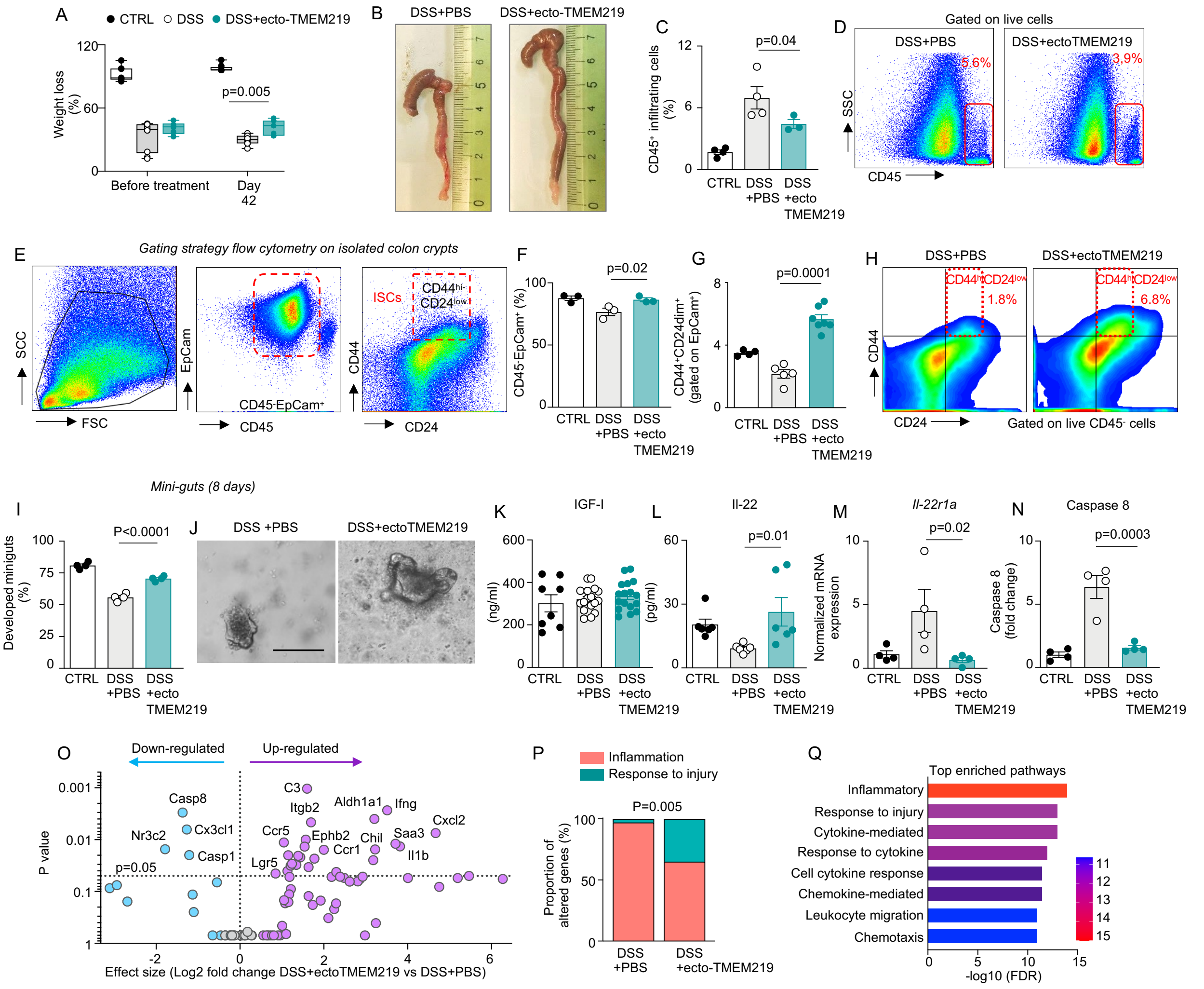

(A). Weight loss (%) measured at baseline (before treatment) and at day 42 in control, DSS+PBS and DSS+ecto-TMEM219 treated mice, (n=10). **(B)**. Representative pictures of colons harvested at day 42 in DSS+PBS and DSS+ecto-TMEM219 treated mice. Scale bar 1 cm. **(C, D)**. Flow cytometric analysis of CD45$^+$ leucocytes analyzed in colonic samples of DSS+PBS and DSS+ecto-TMEM219 treated mice (Day 42, n=4). **(E)**. Flow plots showing the gating strategy (untreated mice), employed for the analysis of intestinal stem and progenitor cells (CD44^{hi}CD24^{low} cells) and intestinal epithelial cells (CD45$^-$EpCam$^+$ cells). **(F)**. Flow plot and bar graph depicting percentage of CD45$^-$EpCam$^+$ cells measured in colon of mice receiving chronic DSS+PBS or DSS plus ecto-TMEM219 (n=3). **(G, H)**. Flow cytometric analysis of EpCam$^+$CD44^{hi}CD24^{low} intestinal stem cells analyzed in colon of DSS+PBS, DSS+ecto-TMEM219 treated and untreated mice (Day 42, n=5-7). **(I, J)**. Development of 8-days mini-guts obtained from colons of control, DSS+PBS and

DSS+ecto-TMEM219 treated mice (n=4). Original magnification 20×. Scale bar 100 µm. **(K, L)**. IGF-I serum levels measured by ELISA and IL-22 serum levels measured by proQuantum assay in untreated mice and in those receiving chronic DSS+PBS or DSS+ecto-TMEM219 (n=6). **(M)**. Il-22-receptor mRNA relative expression in colon of untreated mice and in those receiving chronic DSS+PBS or DSS+ecto-TMEM219 (n=4/group). **(N)**. Caspase 8 measured by ELISA and expressed as fold change in DSS+ecto-TMEM219-treated and DSS+PBS-treated mice as compared to untreated mice (n=4/group). **(O)**. Volcano plots showing up- and down-regulated genes found in the colonic transcriptome analysis of mice receiving DSS+PBS or DSS+ecto-TMEM219 (n=4). **(P)**. Proportion of genes with altered expression in colon of DSS+PBS as compared to DSS+ecto-TMEM219 and grouped according to their role in inflammation or damage response. **(Q)**. Barplot representing the top enriched signaling pathways found in N. Data are expressed as mean ± standard error of the mean. One-way ANOVA with Sidak's post-hoc test. mRNA expression was normalized to *Hprt*.

Supplementary Figure 8. **Tmem219 genetic deletion in ISCs ameliorates DSS-mediated acute colitis *in vivo* in a preventive setting**.

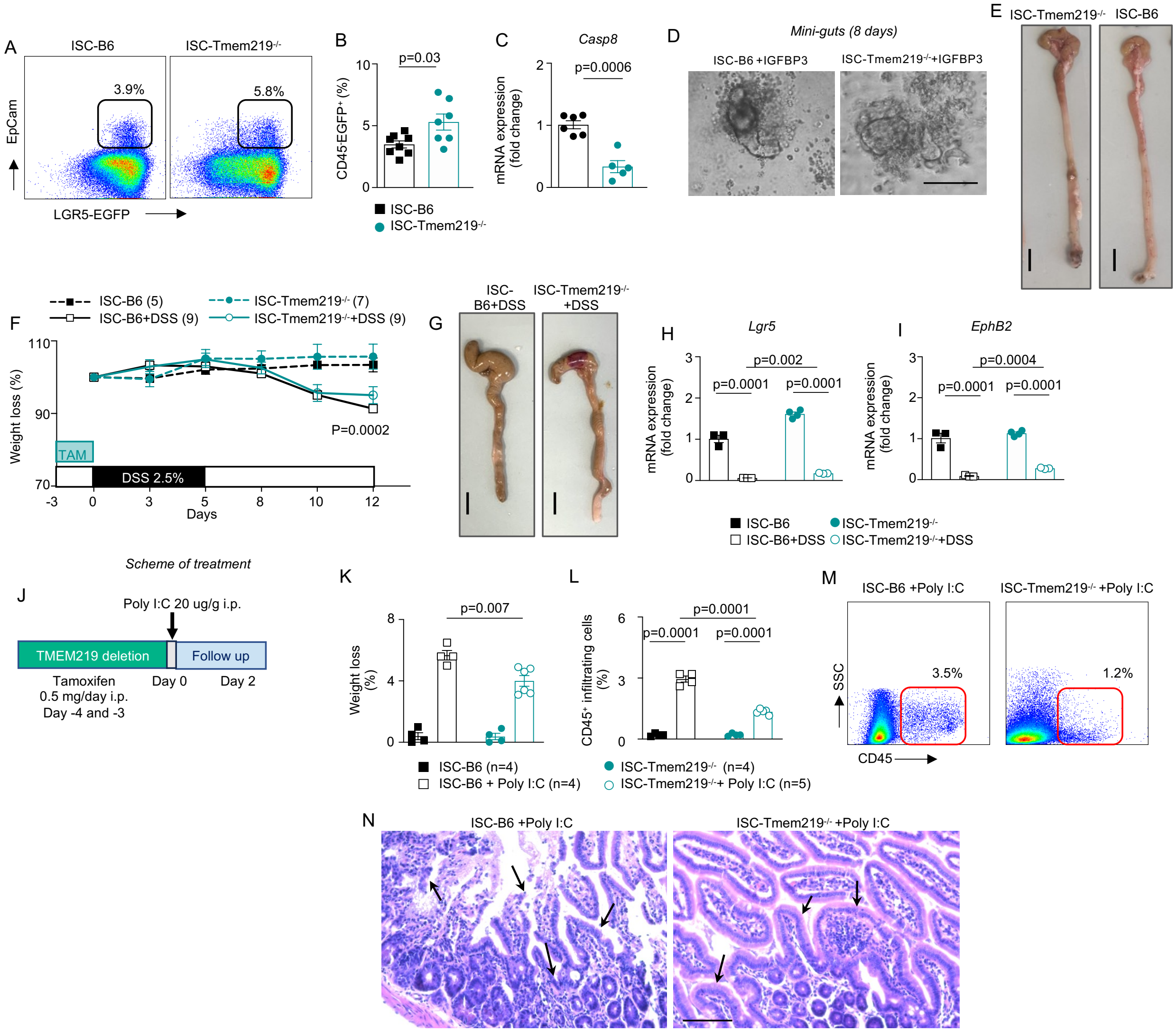

(A, B). Flow plot and bar graph quantifying the percentage of $CD45^{-}EGFP^{+}$ isolated from intestine of $Tmem219^{fl/fl}$ EGFP-$Lgr5^{cre}$ in which tamoxifen was injected, the ISC-$Tmem219^{-/-}$ (n=7), or not injected, the ISC-B6 mice (n=8). **(C)**. Bar graph representing *Casp8* mRNA expression quantified in $CD45^{-}EGFP^{+}$ cells of ISC-$Tmem219^{-/-}$ (n=5) and ISC-B6 mice (n=6). **(D)**. Representative pictures showing *ex vivo* generated 8-day mini-guts from crypts of ISC-$Tmem219^{-/-}$ mice and of ISC-B6 control cultured with IGFBP3 50 ng/ml. Original magnification 20×. Scale bar 100 µm. **(E)**. Representative pictures of colon isolated from ISC-$Tmem219^{-/-}$ and ISC-B6 mice. Scale bar 1 cm. **(F)**. Line graph depicting weight loss measured in ISC-$Tmem219^{-/-}$ and ISC-B6 mice receiving or not DSS 2.5% in a prevention setting (n=9, n=5 untreated controls). **(G)**. Representative pictures of colon isolated from ISC-$Tmem219^{-/-}$ and ISC-B6 mice receiving DSS in the preventive setting. Scale bar 1 cm. **(H, I)**. Bar graph showing normalized mRNA expression of ISC marker *EphB2* and *Lgr5* analyzed by qRT-PCR in samples of ISC-$Tmem219^{-/-}$ mice and of ISC-B6 control

receiving DSS in a preventive setting (n=3-4/group). **(J)**. Experimental design of the Poly I:C enteritis model conducted in the ISC-Tmem219$^{-/-}$ mice. **(K)**. Bar graph quantifying weight loss (%) at 36 hours in ISC-Tmem219$^{-/-}$ mice injected with Poly I:C (20 µg/g, n=6) as compared to ISC-B6 control (n=4). **(L-M)**. Bar graph and flow plots representing infiltrating CD45^{+} cells measured in intestinal samples of ISC-Tmem219$^{-/-}$ (n=5) and ISC-B6 mice (n=4) injected with Poly I:C. (N). Histological anecdotical pictures (H&E) of intestinal samples obtained from ISC-Tmem219$^{-/-}$ and ISC-B6 mice treated with Poly I:C. Arrows highlight crypts morphology, infiltration and edema. Original magnification 20×. Scale bar 500 µm. Data are expressed as mean ± standard error of the mean unless otherwise reported. mRNA expression was normalized to *Hprt*. Two-sided t-test Student t test, Two-way ANOVA and One-way ANOVA with Sidak's post hoc test were used for statistical analysis. ISC-B6, Tmem219^{flfl}EGFPLgr5^{cre} in whom cre was not activated by tamoxifen injection; ISC-Tmem219$^{-/-}$, mice in which Tmem219 was genetically deleted in Lgr5 cells; qRT-PCR, quantitative real-time polymerase chain reaction; Arb, arbitrary.

Supplementary Figure 9. Tmem219 genetic deletion in ISCs ameliorates DSS-mediated acute colitis *in vivo* in a curative setting.

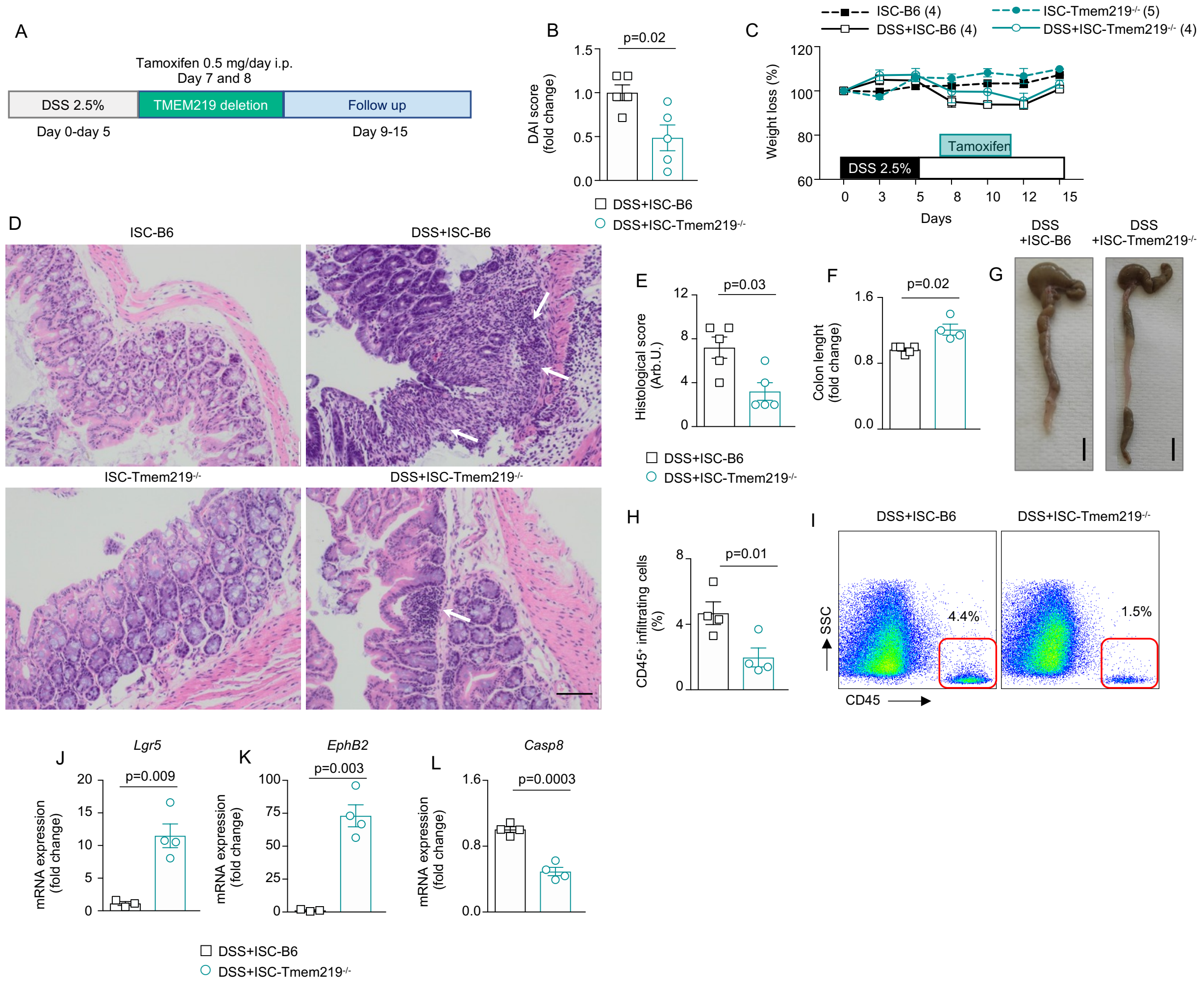

(A). Experimental design of the DSS acute curative model conducted in the ISC-Tmem219$^{-/-}$ mice. **(B, C)**. DAI score and weight loss measured in ISC-Tmem219$^{-/-}$ and ISC-B6 mice receiving or not DSS 2.5% in a curative setting (n=4 and 5 respectively). **(D, E)**. Histological score and representative morphological pictures (H&E) of intestinal samples obtained from mice as described in B. Arrows highlight inflammation and leukocytes infiltration. Original magnification 20×. Scale bar 500 µm. **(F, G)**. Colon length fold increase, with representative pictures in G, quantified in ISC-Tmem219$^{-/-}$ mice and in ISC-B6 control (n=4 and 5 respectively). **(H, I)**. Bar graph and flow plots representing infiltrating CD45^{+} cells measured in samples of ISC-Tmem219$^{-/-}$ and ISC-B6 mice in the curative setting, (n=4/group). **(J, K, L)**. Normalized mRNA expression of ISC marker *EphB2* and *Lgr5* and of *Casp8* analyzed by qRT-PCR in ISC-Tmem219$^{-/-}$ and ISC-B6 control receiving DSS, (n=4/group). Data are expressed as mean ± standard error of the mean unless otherwise reported. mRNA expression was normalized to *Hprt*. Two-sided Student t test, Mann-Whitney test. ISC-B6, Tmem219^{flfl}EGFPLgr5^{cre} in whom cre was not activated by tamoxifen injection; ISC-Tmem219$^{-/-}$, mice in which Tmem219 was genetically deleted in Lgr5 cells.

Supplementary Figure 10. IGFBP3-TMEM219 *in vitro* binding studies.

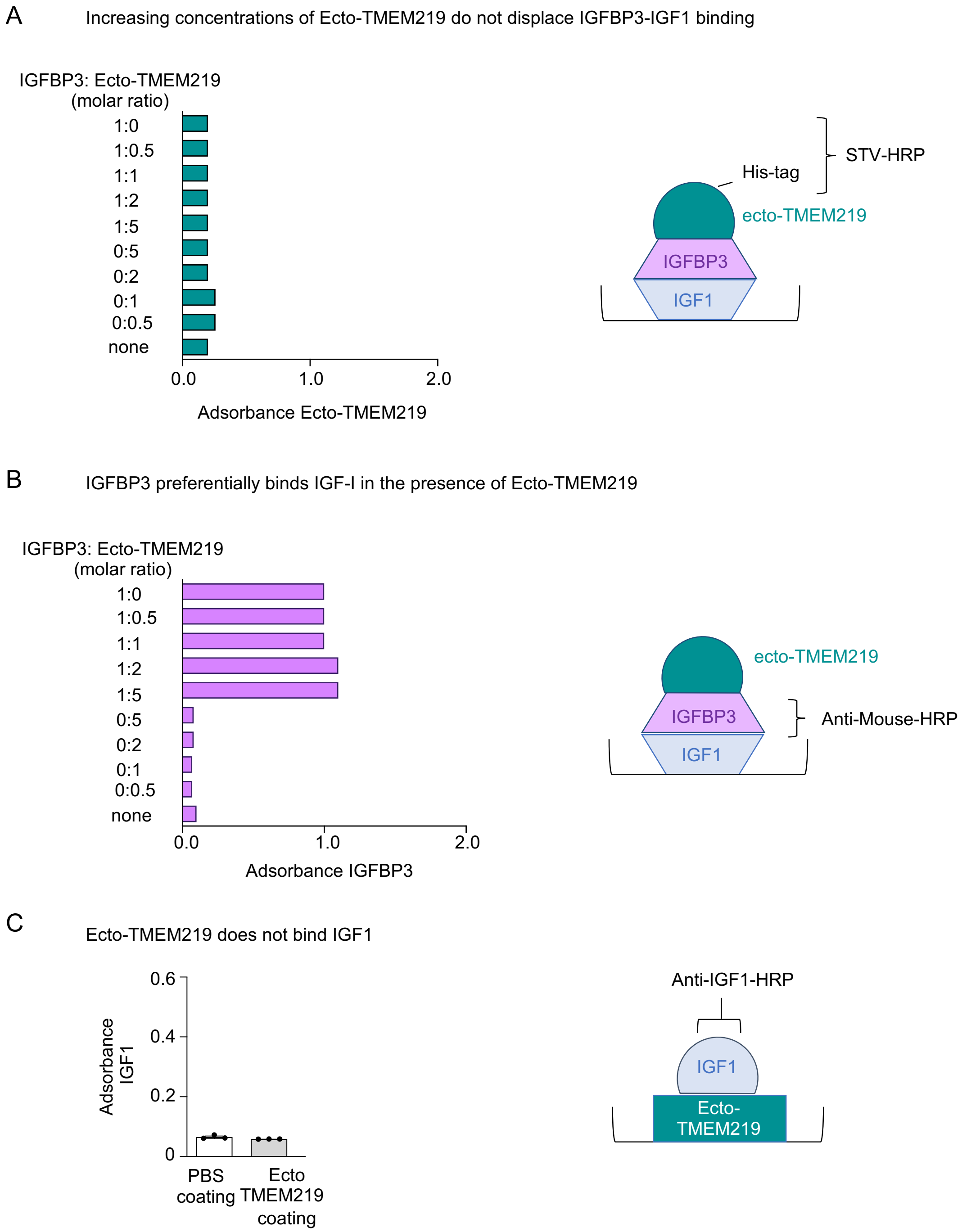

(A). Competitive binding study showing that increasing concentrations of ecto-TMEM219 do not displace the IGFBP3-IGF-I binding in a competitive ELISA assay. Absorbance of ecto-TMEM219 was measured. **(B)**. Competitive binding study showing that, in the presence of ecto-TMEM219, IGFBP3 preferentially binds IGF-I. Absorbance of IGFBP3 was measured. **(C)**. Binding study showing that ecto-TMEM219 does not bind IGF-I through ELISA. Absorbance of IGF-I was measured.

References

1. Langmann T, Moehle C, Mauerer R, Scharl M, Liebisch G, Zahn A, et al. Loss of detoxification in inflammatory bowel disease: dysregulation of pregnane X receptor target genes. *Gastroenterology.* 2004;127(1):26-40.
2. Jung P, Sato T, Merlos-Suarez A, Barriga FM, Iglesias M, Rossell D, et al. Isolation and in vitro expansion of human colonic stem cells. *Nat Med.* 2011;17(10):1225-7.
3. Baker AM, Graham TA, Elia G, Wright NA, and Rodriguez-Justo M. Characterization of LGR5 stem cells in colorectal adenomas and carcinomas. *Sci Rep.* 2015;5:8654.
4. Sun S, Xue J, Guo Y, and Li J. Bioinformatics analysis of genes related to ferroptosis in hepatic ischemia-reperfusion injury. *Front Genet.* 2022;13:1072544.
5. Mostafavi S, Ray D, Warde-Farley D, Grouios C, and Morris Q. GeneMANIA: a real-time multiple association network integration algorithm for predicting gene function. *Genome Biol.* 2008;9 Suppl 1(Suppl 1):S4.
6. D'Addio F, La Rosa S, Maestroni A, Jung P, Orsenigo E, Ben Nasr M, et al. Circulating IGF-I and IGFBP3 Levels Control Human Colonic Stem Cell Function and Are Disrupted in Diabetic Enteropathy. *Cell stem cell.* 2015;17(4):486-98.
7. Barker N, van Es JH, Kuipers J, Kujala P, van den Born M, Cozijnsen M, et al. Identification of stem cells in small intestine and colon by marker gene Lgr5. *Nature.* 2007;449(7165):1003-7.
8. Dieleman LA, Ridwan BU, Tennyson GS, Beagley KW, Bucy RP, and Elson CO. Dextran sulfate sodium-induced colitis occurs in severe combined immunodeficient mice. *Gastroenterology.* 1994;107(6):1643-52.
9. D'Alessio S, Correale C, Tacconi C, Gandelli A, Pietrogrande G, Vetrano S, et al. VEGF-C-dependent stimulation of lymphatic function ameliorates experimental inflammatory bowel disease. *J Clin Invest.* 2014;124(9):3863-78.
10. D'Addio F, Maestroni A, Assi E, Ben Nasr M, Amabile G, Usuelli V, et al. The IGFBP3/TMEM219 pathway regulates beta cell homeostasis. *Nat Commun.* 2022;13(1):684.
11. Zhou R, Wei H, Sun R, and Tian Z. Recognition of double-stranded RNA by TLR3 induces severe small intestinal injury in mice. *J Immunol.* 2007;178(7):4548-56.
